# TDP-43: [GU]-ardian of the transcriptome

**DOI:** 10.1186/s13024-026-00944-2

**Published:** 2026-05-14

**Authors:** Irika R. Sinha, Abigail L. Atkinson, Katherine E. Irwin, Jonathan P. Ling, Philip C. Wong

**Affiliations:** 1 Department of Neuroscience, Johns Hopkins School of Medicine, Baltimore, MD USA; 2 Department of Pathology, Johns Hopkins School of Medicine, Baltimore, MD USA; 4 Present Address: Allen Institute, WA Seattle, USA; 3 Medical Scientist Training Program, Johns Hopkins School of Medicine, Baltimore, MD USA

## Abstract

TDP-43 is a ubiquitously expressed, primarily nuclear DNA/RNA-binding protein implicated in neurodegenerative diseases including amyotrophic lateral sclerosis (ALS), frontotemporal dementia (FTD), and Alzheimer’s disease (AD). In this review, we examine the structure and regulation of TDP-43, how these features influence its localization and functional activity, and how their disruption may contribute to disease. Among TDP-43’s diverse functions, splicing repression of nonconserved RNA sequences termed cryptic exons has emerged as especially central to human disease. TDP-43 nuclear depletion and cytoplasmic aggregation are well-established pathological features in affected neurons and glia of neurodegenerative diseases, and accumulating evidence suggests that loss of TDP-43-mediated splicing repression occurs presymptomatically in disease. Advances in RNA-sequencing have enabled systematic identification of cryptic exon inclusion as a sensitive marker of TDP-43 dysfunction. Here, we synthesize current knowledge of TDP-43 biology and curate datasets from human tissues and experimental models, focusing on cryptic splicing to provide a resource for leveraging cryptic exon biology to better understand, detect, and target TDP-43 dysfunction.

## Introduction

Originally identified as a transcriptional repressor of human immunodeficiency virus 1 (HIV-1) [1], transactive response DNA binding protein 43 (*TARDBP,* TDP-43) is a ubiquitously expressed, primarily nuclear protein of the heterogeneous nuclear ribonucleoprotein (hnRNP) family which has since been implicated in a wide range of diseases [2–4]. Early research in the 2000s suggested it played an important role in splicing regulation [5–7], and it was identified in 2006 as a major component of urea-soluble, ubiquitinated cytoplasmic inclusions in end-stage cases of amyotrophic lateral sclerosis and frontotemporal dementia (ALS-FTD) [8–12]. Between 2006 and 2015, TDP-43’s role in splicing regulation was further characterized, and in 2015, its role in suppressing cryptic exons (CEs) was uncovered and confirmed to occur in cases of ALS-FTD exhibiting TDP-43 pathology [13–20]. In addition to ALS-FTD, TDP-43 pathology and associated cryptic exons have been observed in other disorders, such as limbic-predominant age-related TDP-43 encephalopathy (LATE) [21–23], inclusion body myositis (IBM) [24–26], and Alzheimer’s disease (AD) [23, 27–33], among others [34–43].

While TDP-43 is not the primary pathological species in many of these diseases, its widespread occurrence in neurodegeneration and other diseases underscores a need to better understand its biological roles and pathogenic mechanisms. Current literature has explored pathological TDP-43 as a biomarker by investigating levels of phosphorylated or vesicle-enclosed forms of the protein [44–48], variant forms of TDP-43 or anti-TDP-43 autoantibodies [49–52], and, most recently, targets of TDP-43 splicing regulation [53–56] in patients with ALS-FTD.

Although studies of dysregulation and dysfunction have deepened understanding of TDP-43 pathophysiology, the biology of TDP-43 and its variation across cellular compartments remain poorly understood. Developing therapeutics focused on treating TDP-43 dysfunction include application of antisense oligonucleotides (ASOs) to prevent aberrant splicing of specific TDP-43 targets. In this review we focus on TDP-43’s role as a splicing repressor and the consequences of its dysfunction, with the goal of highlighting aspects of its biology that could clarify disease mechanisms and inform targeted therapeutics strategies for TDP-43 proteinopathy.

## Structure of TDP-43

### Genetics of *TARDBP* and its translation product TDP-43

*TARDBP* is an evolutionarily conserved gene located on chromosome 1 of the human genome [57, 58]. Alternative splicing of the gene primarily occurs in exon 6, at the 3’ end of the gene, and some truncated forms of the translated protein have been characterized in the context of various pathologies [57, 59–61]. *TARDBP* is essential for viability as constitutive deletion of mouse *Tardbp* is lethal during both embryonic development and adulthood [62–64]. Additionally, TDP-43 nuclear depletion and loss of RNA-binding abilities leads to cellular toxicity in various model systems, highlighting the roles of localization and RNA-binding in protein functionality [65, 66].

**Fig. 1:**
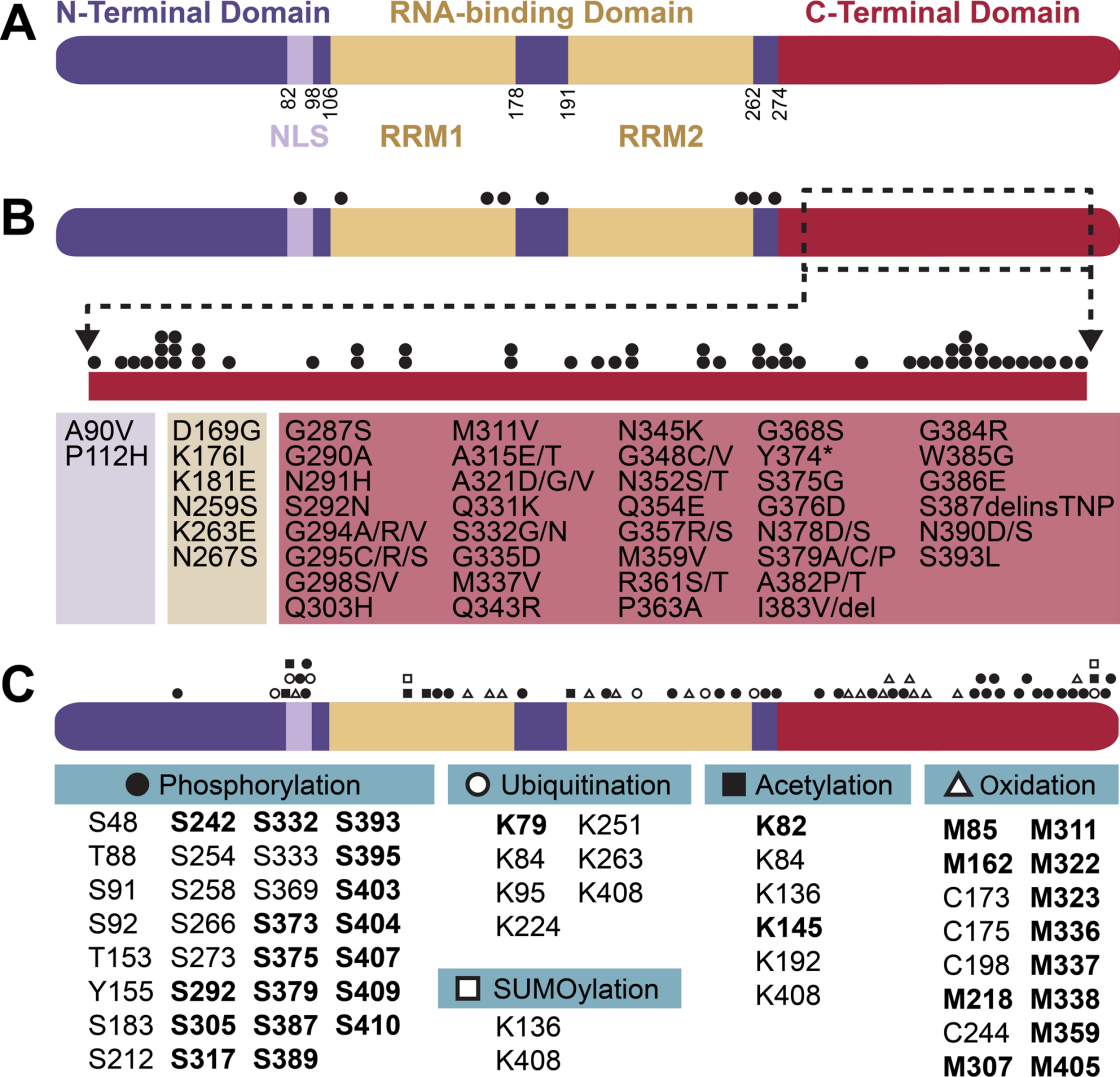
TDP-43 protein structure and modifications. **A**. TDP-43 contains three main domains: the N-terminal, RNA-binding, and C-terminal domains. A nuclear localization signal (NLS) and two RNA recognition motifs (RRMs) are contained within the N-terminal and RNA-binding domains, respectively. The C-terminal domain of TDP-43 is a glycine-rich, aggregation-prone region. **B**. A map of the disease-associated genetic variants in *TARDBP* which lead to missense or nonsense mutations is depicted with each circle representing a unique known mutation in the protein. The majority are located within the C-terminal domain. The listed variants have been identified in patients with varied neurologic disorders, including ALS-FTD and PD [66–136]. However, the pathogenicity of some variants remains uncertain and/or debated (e.g., A90V). **C. **TDP-43 has many potential sites for post-translational modifications, including 64 total serines, threonines, and tyrosines that could possibly be phosphorylated. A subset of sites have been confirmed as modified in disease cases (bolded). Others, tested in vitro, are suggested to impact the localization, function, and/or polymerization of TDP-43 [137–160]

Full-length TDP-43 is a 414 amino acid RNA-binding protein (RBP) with a molecular weight of 43 kDa and four major structural domains (Fig. 1A). These include an N-terminal region with a nuclear localization signal (NLS), two RNA recognition motifs (RRMs), and a glycine-rich C-terminal domain with a glutamine and asparagine-rich subdomain often described as an intrinsically disordered region (IDR) or prion-like domain (PrLD) [137, 161]. X-ray crystallography and cryo-EM studies of TDP-43 as domain fragments and as complexed fibrils have pieced together a protein made up of fixed domains and flexible linker regions [162–170].

The N-terminal region of TDP-43, containing the NLS, is crucial in maintaining TDP-43 localization to the nucleus and, thus, mRNA splicing regulation. After TDP-43 translation in the cytoplasm, the NLS recruits chaperone proteins such as importin-ɑ1 and importin-β1 to transport mature TDP-43 into the nucleus through RAN-regulated mechanisms [65, 171–176]. Mutation or removal of the NLS disrupts TDP-43 localization, with recent work suggesting mutations or acetylation of nearby amino acids can also induce TDP-43 mislocalization [65, 67, 138, 139, 177, 178].

Successive tandem RRMs follow the N-terminal region of TDP-43 and compose the RNA-binding domain. This domain is necessary for the specific recognition of and binding ability to uracil-guanine [UG]_n_ repeats [15, 66, 179–183]. Without the RRMs, TDP-43 is unable to recognize its targets or prevent CE inclusion. As an aside, dimeric TDP-43 has also been shown to bind single-stranded DNA via the RRMs, the implications of which remain an active area of study [180, 184–186].

Finally, while there is evidence suggesting the C-terminal domain contributes to splicing regulation [187, 188], it is predominantly involved in modulating protein-protein interactions [189]. This domain is crucial for TDP-43’s ability to form liquid droplets in cells through liquid-liquid phase separation (LLPS) and to interact with other proteins in structures such as stress granules [189–192]. Furthermore, mutations in *TARDBP* associated with neurodegenerative diseases primarily occur within this aggregation-prone C-terminal domain of the protein [193, 194].

*TARDBP* contains many known mutations linked to ALS-FTD [68–73], as well as some mutations associated with other neurodegenerative disorders such as AD [74, 195, 196] (Fig. 1B). Over 80 ALS-causing mutations in TDP-43 have been described in human populations thus far, and they are found in 3–5% of patients with familial ALS and fewer than 1% of patients with sporadic ALS [75]. Most of these mutations are missense mutations located in the C-terminal domain of TDP-43, and some have been correlated with increased tendencies for aggregation [74, 193, 197]. However, other models of these mutations display phenotypes of decreased aggregation (e.g., D169G and K263E), increased half-life (e.g., D169G, K263E, G298S, A315T, M337V), or increased degradation rate (e.g., R361S) [68, 69, 71, 74, 76–79, 198–203].

The observed effects of these mutations vary significantly depending on model and context, and splicing phenotypes remain an active area of research. For example, a cell culture model of M337V indicates disrupted polyadenylation patterns, and a mouse model displays missplicing of known TDP-43 targets [204]. In contrast to the many known C-terminal mutations, disease-associated mutations in the N-terminal NLS or RRM domains are much rarer, likely due to essential roles of these regions in TDP-43 localization and function [66, 202]. One example, however, is K263E, which induces measurable polyadenylation changes in a cell culture model [205].

### Post-translational modifications (PTMs)

Research on TDP-43 PTMs in healthy contexts is limited, with most studies focused on modifications within disease [137, 194, 206, 207]. TDP-43 aggregates in ALS-FTD are found composed of highly ubiquitinated, hyperphosphorylated, and truncated proteins. Cleavage of the protein, potentially by apoptosis-related protease Caspase-3 or calcium-dependent cysteine protease Calpain [208–214], leads to C-terminal, truncated protein fragments which lack the NLS and are mislocalized to the cytoplasm [8, 11, 140, 141, 198, 215]. However, the consequences of these PTMs are still debated. This section focuses on mutations that result in altered splicing phenotypes, often measured using a CFTR minigene splicing assay [216].

Hyperphosphorylation of TDP-43, particularly at S369, S379, S403/404, and S409/410, is seen in a vast majority of patient tissues with TDP-43 proteinopathy (Fig. 1C) [140–145, 217]. While some suggest that phosphorylation is closely tied to aggregation [218], others propose that it could serve a protective purpose and preserve TDP-43’s ability to undergo LLPS [219–221]. Phosphomimetics of TDP-43 indicate phosphorylation at S48, T153, Y155, and both hyper- and hypo-phosphorylation of the C-terminal domain appear to reduce TDP-43 splicing regulation capabilities [146, 222, 223], and thus may increase cryptic exon inclusion. Cellular compartmentalization of kinases and phosphatases targeting TDP-43, as well as site-specificity likely play a large role in effect variance [145, 147].

Similarly, known mechanisms and functions of TDP-43 polyubiquitination remain limited, although many putative ubiquitination sites have been identified in TDP-43 (Fig. 1C) [148]. While ubiquitination is classically linked to protein degradation pathways via the proteasome [224], it can also be important in protein signaling and localization. This functional variation is reflected in TDP-43 biology. Some research suggests ubiquitination of TDP-43 is associated with increased protein turnover [225], but others have found polyubiquitination linked to increased cytosolic aggregation instead [226, 227]. This may be linked to the variety in polyubiquitin chain structures which allow for more complex protein regulation functions [228–230].

Other PTMs associated with TDP-43 include acetylation, SUMOylation, and cysteine oxidation (Fig. 1C) [137, 194]. Lysine acetylation of TDP-43 is linked to impaired protein function and has been identified in tissue from ALS cases [149]. Acetylation of Lys-136 or Lys-145, located in the RRMs, has been associated with increased cytosolic aggregation and a loss of splicing function [138, 149, 231–233]. Acetylation of Lys-82 or Lys-84, near and in the NLS, appear to disrupt TDP-43 nuclear import by inhibiting interaction with importin ɑ1 [138, 139]. In all these cases, acetylation of TDP-43 leads to mislocalization and would likely reduce its splicing regulation capabilities accordingly. Additionally, although acetylation of residues seems to play a role in inducing export, its impact is likely still highly context-dependent, and further work in this area is needed to better understand its effects on TDP-43 function and role in disease.

In contrast, although SUMOylation has been observed in aggregation models, it is also recognized for its protective effects on cellular health and some results indicate it may be necessary for appropriate splicing of TDP-43 targets [150, 151, 234–236], such as *MADD*, which contains a cryptic exon [152]. Finally, oxidation of TDP-43 has been suggested as part of an oxidative stress response with cysteine oxidation leading to disulfide bridge formation and inter- and intra- protein crosslinking [153, 237, 238]. Both mutation and oxidation of RRM domain cysteine residues led to increased aggregation of TDP-43, and oxidation was also found to result in loss of nucleic acid-binding capabilities [154, 239]. This suggests oxidation may also lead to loss of TDP-43 splicing repressor activity due to an inability to bind its RNA targets.

## Regulation of TDP-43

### RRMs and 3’ Untranslated Region (UTR) are essential for autoregulation

A notable method of TDP-43 autoregulation involves the binding of the translated protein to its own 3’ UTR sequence. High levels of cellular TDP-43 increase levels of bound pre-mRNA transcript, resulting in altered splicing and decreased transcript stability. As a result, the transcript is degraded and TDP-43 is downregulated [240–244]. This form of autoregulation requires functional RRM and C-terminal domains, as both are needed for TDP-43 to bind the autoregulatory site. During this process, shorter forms of TDP-43 (sTDP-43) lacking the C-terminal domain and with a putative nuclear export signal are generated. Improper accumulation of sTDP-43 can further induce aggregation and impairment of full-length TDP-43. These truncated forms of TDP-43 are mislocalized and unable to regulate splicing activity within the nucleus, likely contributing to transcriptome dysregulation. Although this isoform is normally rapidly degraded, cytoplasmic deposits have been detected within tissue from ALS cases, indicating potential regulatory failures capable of inducing or worsening TDP-43 dysfunction [60, 61].

This autoregulatory mechanism likely also plays a role in TDP-43 happlosufficiency and the rarity of TDP-43 RRM mutants. The homozygous knockout of *Tardbp* is embryonically lethal in mouse models, possibly due to the immensity of dysregulated targets and the subsequently compromised proteome. However, heterozygous *Tardbp*^+/-^ mice are generally phenotypically normal at early ages and express levels of TDP-43 protein similar to *Tardbp*^+/+^ littermates [62–64, 245, 246]. Furthermore, the toxicity of RRM deficiency [247] may be associated with this autoregulatory mechanism. Inability of the protein to bind to the autoregulatory domain would prevent appropriate reduction of protein levels. As overexpression of TDP-43 is toxic in a dose-dependent manner, increasing amounts of the protein due to failed autoregulation would also cause early lethality [248].

### RNA-binding dependency of TDP-43 nuclear localization

Nuclear localization of TDP-43 is dependent on NLS-mediated nuclear import and diffusion-mediated passive export through the nuclear pore complex (NPC), as evidenced by mutant NLS resulting in cytoplasmic accumulation and concentration-dependent aggregation of TDP-43. It is worthwhile to note that experimentally increasing its size with the addition of a protein tag can also increase nuclear retention and must be considered when designing experiments to monitor TDP-43 localization [171–173, 249–251]. Additionally, physiological concentrations of [UG]_n_-rich RNA play a role in nuclear retention of TDP-43. Nuclear binding of TDP-43 to [UG]_n_-rich RNA molecules creates an RNA-protein complex too large to diffuse through the NPC. Multivalent binding of the protein can increase the size of this complex even further and allow for cooperative assemblies of TDP-43 [252]. In contrast, a reduction of nuclear RNA through RNAse treatment or RNA polymerase inhibition increases the rate of export. Similarly, the binding of TDP-43 to short RNA molecules rather than mRNA targets of a much larger size, creates a complex small enough to pass through the NPC [253–255]. While these results are measured in vitro, they suggest TDP-43 efflux and reduction in nuclear splicing functions can be induced by failures in RNA lifecycle mechanisms.

### Protein modulators of TDP-43 activity

Although TDP-43 alone is sufficient to repress its associated cryptic exons, it interacts with a variety of protein partners and forms complexes within the cell that likely influence its RNA binding ability, localization, and splicing regulatory activity. Some interactions also seem RNA-binding dependent, further complicating TDP-43 homeostasis [256]. Still, many putative binding partners have been identified in normal, stressed, and aggregated conditions [256–260], although the exact consequences of these interactions remain under study. As previously mentioned, the C-terminal domain of TDP-43 is responsible for many of its protein-protein interactions, and removal of the domain can cause splicing impairment in model systems [187, 197, 216, 261, 262].

One known interactor of TDP-43 is itself. Under physiological conditions, the N-terminal domain of TDP-43 likely mediates dimerization or oligomerization, which is necessary for splicing activity. However, this normal multimerization, which suppresses aggregation, is impaired in disease [180, 263–267]. Instead, TDP-43 aggregates and this leads to insoluble, cytosolic inclusions that can be associated with other proteins as identified in end-stage tissues [185, 268–271].

Several of TDP-43’s known interactors are similarly associated with the RNA life cycle and regulation, and may play a role in modulating the splicing activities of TDP-43. For example, other hnRNP protein members are known TDP-43 binding partners [181, 187, 272]. However, knockdown of hnRNP A1, hnRNP A2B, or hnRNP L alone is insufficient to induce cryptic splicing in the tested target, cryptic *UNC13A* [273], suggesting that TDP-43-mediated splicing regulation does not rely on those proteins. However, overexpression of these proteins reduces cryptic *UNC13A* transcripts in the absence of TDP-43, and hnRNP L levels inversely correlate with cryptic *UNC13A* in cortex of ALS-FTD cases, suggesting they may partially compensate for TDP-43 loss [273]. Additionally, cross-Linking and immunoprecipitation (CLIP) analyses indicate that other RBPs, including U2AF65, can bind cryptic exon sites and may therefore compete or interfere with TDP-43 binding [273, 274]. For example, TDP-43 is able to form a complex with FUS/TLS and stabilize *HDAC6* mRNA, suggesting that FUS/TLS may be able to modify the RNA regulation properties of TDP-43 [261, 275]. However, as this complex’s affinity appears to strengthen with mutations in TDP-43, and both proteins occur in pathological aggregates, it may be more pathogenic in nature and cause increased cell destabilization [197, 276]. Indeed, elevated levels of HDAC6 decreases autophagic pathway function while inhibition of HDAC6 in a patient cell model appears to reduce levels of cytoplasmic, insoluble TDP-43 [233, 276].

Proteome and predictive TDP-43 proteome analysis also highlights some binding partners that are impacted by TDP-43 splicing regulation [259]. For example, USP10 is predicted to interact with TDP-43 but recent preprints suggest its transcript also contains a TDP-43-mediated CE [277–279]. Additionally, USP10 plays a role in preventing cytoplasmic aggregation of TDP-43 and may be able to modulate levels of TDP-43 splicing [280, 281]. However, these interactions will likely be diminished in the context of TDP-43 loss, as some *USP10* transcript will include the cryptic exon and no longer encode the functional protein.

Genome-wide association studies (GWAS) also suggest that genetic variations in other genes can impact TDP-43 pathology. While the functional consequences of the variation on gene expression or function are not always known, the implicated genes may affect TDP-43 activity. For example, the *UNC13A* gene contains single nucleotide polymorphisms (SNPs) associated with ALS-FTD near a TDP-43-regulated cryptic exon [282–286]. Two of these SNPs, in the context of TDP-43 depletion, lead to increased cryptic transcript in vitro and are associated with increased cryptic *UNC13A* burden in ALS-FTD and AD-TDP cases [32, 33, 285–287]. GWAS also suggests variants in *TMEM106B* are linked to FTLD-TDP and TDP-43 proteinopathy cases and associated with worsened TDP-43 aggregation [288–292]. Additionally, *APOE*, a well-known AD risk gene [293], also contains variants associated with TDP-43 proteinopathy [292, 294–297]. A study of familial FTLD-MND found that *APOE* genotype was associated with severity and distribution of TDP-43 pathology. It further identified complexes containing both ApoE and TDP-43 in brain tissue, raising the possibility of a direct molecular interaction between ApoE and TDP-43 [298]. Larger studies have also shown that the APOE ε4 allele is associated with an increased frequency of TDP-43 pathology, including in AD [31].

### Cellular stress can alter TDP-43 localization and activity

Cytoplasmic mRNA can be regulated through sequestration in “storage unit” membraneless organelles (MLOs) known as processing bodies, or P-bodies. These P-bodies will then release mRNA for translation as needed or coordinate transcript degradation [299]. TDP-43 may play a role in indirectly regulating the formation of these transitory units, possibly through the direct regulation of cytoplasmically accessible P-body components [300]. Under certain types of cellular stress, mRNA may transition to another MLO which facilitates the acute RNA response to the stressor.

Stress granules (SGs) are MLOs in the cytoplasm which contain a combination of mRNAs, proteins, and ribosomal components. They form through LLPS and are a well-known cellular response to short-term stressors. mRNA relocalization to SGs halts translation and decreases cellular energy requirements as they are sorted for continued translation efforts based on necessity [301]. Within stressed conditions, TDP-43 can be found associated with SGs, suggesting its cytoplasmic relocation may be induced, in part, by environmental factors such as oxidative stress [192, 256, 302–305]. Specific stressors, such as oxidative stressor sodium arsenite [149, 153, 181, 306–314], are known to induce TDP-43 loss of function and mislocalization as well, and repeated or consistent cellular stress may increase levels of mislocalized TDP-43 or prevent proper nuclear relocalization. However, removal of the TDP-43 RRMs appear to diminish the ability of the protein to relocalize to the stress granules, suggesting TDP-43 may be recruited as an mRNA-bound RBP. Furthermore, appropriate levels of TDP-43 are necessary for appropriate formation and disassembly of SGs [256, 315, 316].

### Degradation of TDP-43 and impacts on protein homeostasis

Both the ubiquitin-proteasome system and autophagy pathway are involved in turnover of TDP-43, although different forms of TDP-43 may undergo different kinds of degradation. Cellular disruption of either pathway can severely impact TDP-43’s functionality [155, 317–322]. TDP-43 levels also further indirectly influence the autophagy pathway through control of a cryptic exon within the autophagy gene *ATG4B*. This suggests broader impacts on protein homeostasis [323–327]. TDP-43’s role in protein turnover can also be seen under conditions of cellular stress. Accumulation of misfolded proteins and subsequent ER stress can trigger the unfolded protein response (UPR), which then increases mRNA and protein turnover [328]. While the role of TDP-43 in causing UPR remains debated, both ER stress and other cellular stresses influence TDP-43 function and localization, as previously mentioned, and TDP-43 loss can increase protein degradation pathways [329, 330]. Some studies additionally suggest abnormal TDP-43 levels within the mitochondria can induce mitochondrial UPR and further mitochondrial damage [331, 332].

## Functions of TDP-43

### TDP-43 as a splicing regulator and repressor of cryptic exons

**Fig. 2:**
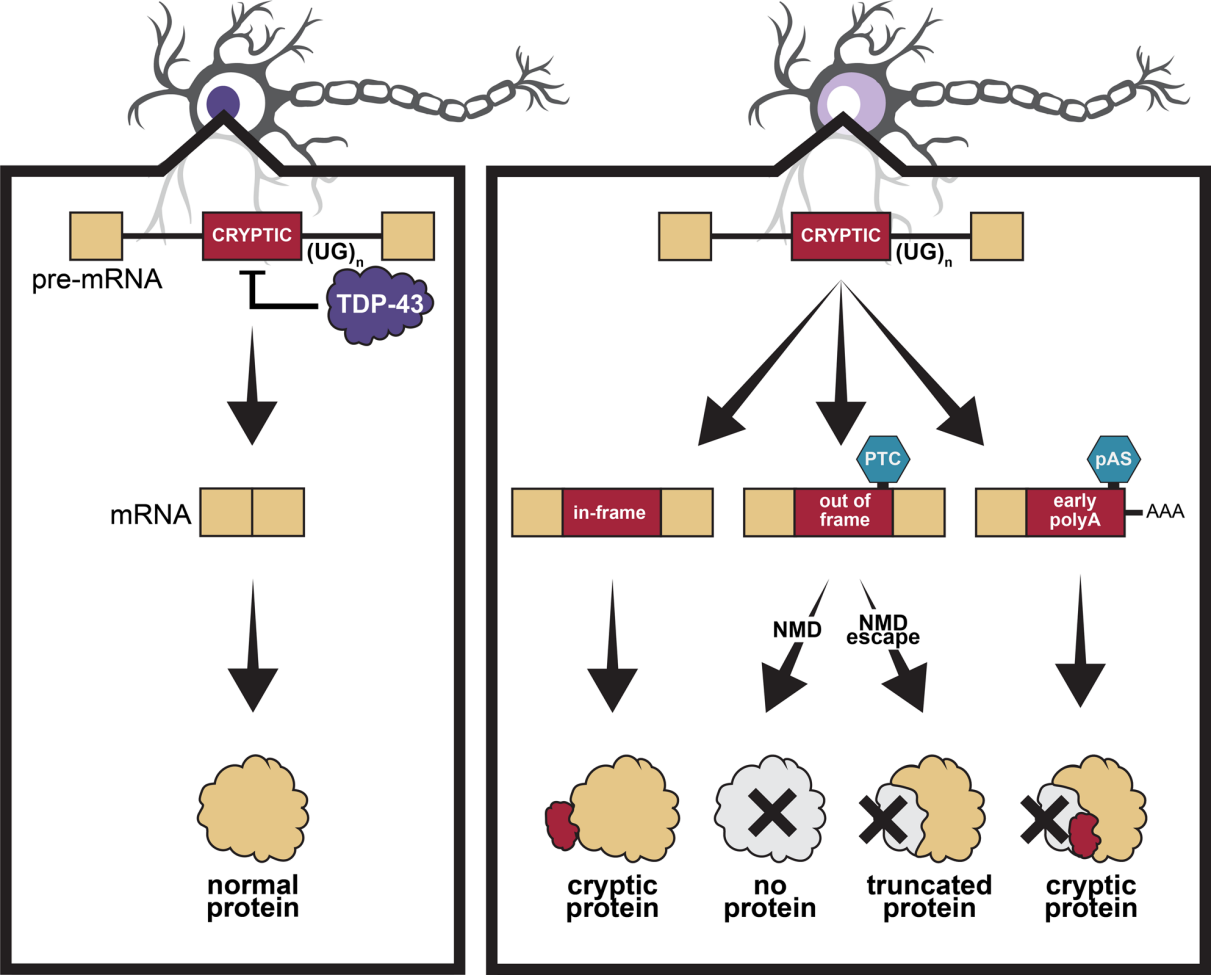
TDP-43 represses the inclusion of cryptic exons. (*left*) TDP-43 localized to the nucleus binds (UG)_n_ repeats in pre-mRNA and prevents nearby cryptic exons from being spliced into mRNA. This leads to the creation of normal protein. (*right*) mislocalized TDP-43 does not bind pre-mRNA in the nucleus, which leads to the inclusion of cryptic exons in mRNA. This cryptic exon can lead to an in-frame or out-of-frame transcript inclusion, or an early polyadenylation site (pAS). An in-frame cryptic exon results in a cryptic protein with a cryptic peptide. Out-of-frame transcripts encode premature termination codons (PTCs), which generally trigger nonsense-mediated decay (NMD) of the transcript and no subsequent protein. Transcripts with PTCs that escape NMD will result in the translation of a truncated protein. Finally, cryptic exons which encode a polyA site (pAS) will result in a truncated protein that likely includes a partial cryptic peptide

TDP-43 plays a key role in regulating splicing and maintaining the transcriptome. It prevents the inclusion of noncanonical “cryptic” exons (CEs) in mature mRNA (Fig. 2) [13, 274, 333, 334]. CEs can be cell-type-dependent and are nonconserved between species due to the low sequence conservation rates of the affected intronic regions [13, 334–337]. TDP-43 preferentially binds to uracil-guanine repeats ([UG]_n_) in pre-mRNA located near the target cryptic splice sites [14, 15, 179, 181, 182]. This dinucleotide motif is the most common microsatellite in the human genome [338], and predicted TDP-43 binding sites near cryptic splice sites are often found close to the 3’ end of the cryptic exon [277, 339]. The structural conditions of the RNA targets, such as the inclusion of a G-quadruplex, may also impact TDP-43 binding abilities [340, 341]. RNA-bound TDP-43 then prevents the spliceosome from recognizing cryptic splice sites through methods such as steric hindrance [342]. Additionally, TDP-43-dependent exon and alternative polyadenylation (APA) regulation occurs in a position-dependent manner; binding of the protein close to splice sites represses those sites while binding further away can enhance inclusion [15, 343].

The presence of cell-type-dependent CEs suggests additional mediators of specificity that could allow TDP-43 to identify the many target sites it is known to repress. TDP-43 is often found in ribonucleoprotein complexes with other species [261, 344]. It is unknown whether TDP-43 function relies on these complexed species for successful repression of CEs, or whether TDP-43 by itself can sufficiently identify its major binding targets. Some studies have investigated the role of other disease-associated RBPs in suppressing TDP-43-associated CEs. While TDP-43 is the primary, shared regulator of these CEs, other proteins may influence the inclusion of CEs. For example, knockout of MATR3, FUS, hnRNP A1, hnRNP A2B, or hnRNP L alone was insufficient to induce cryptic splicing in the tested targets [274, 345]. However, as some studies evaluated individual cryptic exons rather than a panel of targets, the observed effects may be CE-specific. An important consideration is that some TDP-43 targets are themselves RBPs, so missplicing could trigger cascading splicing and proteomic defects. For example, *ELAVL3*, a neuron-specific RBP, contains a TDP-43-regulated cryptic exon which leads to its downregulation [346–348]. *ELAVL3* normally binds U/AU‑rich sequences and regulates alternative splicing and target stability [349–352]. Reduced *ELAVL3* function could destabilize its target mRNAs and further disrupt splicing, potentially creating secondary cryptic exons.

Loss of TDP-43 can cause direct cell death in vitro and instigate pathology in vivo, and surviving cells experience sustained splicing dysregulation which contributes to ongoing cellular stress due to transcriptome and proteome disruption [62–64, 245, 246, 300, 333, 353–361]. TDP-43-associated CEs commonly encode premature termination codons (PTCs) which can increase transcript degradation and, if translated, produce truncated proteins. Several human gene targets affected by this process play important roles in cell function, including *AKT3*, *ATG4B*, *ITGA7*, and *PFKP*, which are involved in cell signaling [362–364], autophagy [365–367], cell interactions [368, 369], and cellular metabolism [370–372], respectively. In zebrafish, although TDP-43 knockout is not embryonic lethal, development is abnormal; fish develop muscle degeneration, insufficient vascularization, and spinal motor neuron mispatterning, leading to increased autophagic flux in neurons likely driven by missplicing and protein misfolding [330, 373]. Although TDP-43 loss does not seem to cause acute neuronal injury, the evidence cited above suggests it plays an upstream role in initiating dysfunction as other cellular stressors compound, such that more susceptible model systems might show faster onset of cell death.

### Regulation of alternative polyadenylation (APA)

Alternative polyadenylation (APA) of RNA transcripts primarily modulates characteristics such as expression levels and mRNA localization when the APA sites exist within the 3’UTR. However, APA sites within the coding region of a transcript can also cause the production of new protein isoforms [374]. Many genes are known to have multiple poly(A) sites within the 3’ UTR and, as mentioned in the “RRMs and 3’ Untranslated Region (UTR) are essential for autoregulation” section, TDP-43 autoregulates its own transcript by modulating usage of its own poly(A) sites (pAS). In addition, it is clear its role in pAS selection is not restricted to its own transcript [343].

As with exons, TDP-43 binding modifies pAS usage in a position-dependent manner (Fig. 2). Binding of the protein near a pAS reduces its usage, while binding further downstream enhances its usage [343]. Furthermore, maintenance of normal poly(A) site selection across the transcriptome requires TDP-43 activity. Without TDP-43, poly(A) site selection has been shown to differ dramatically for hundreds of transcripts in a neuronal context, including *ELP1*, *NEFL*, *TMEM106B* and *SFPQ*, and inclusion of cryptic poly(A) sites within the coding regions of transcripts induces novel protein isoforms [205, 375, 376].

### TDP-43 regulation of the mitochondrial transcriptome

As with other RBPs, TDP-43 has an inherently intrinsically disordered structure which allows the protein in its normal state to pass into and between MLOs [190–192, 377]. TDP-43’s propensity towards LLPS allows the protein to act at sites well outside of the nucleus; by complexing in phase separated groups of proteins and RNA, TDP-43 can be transported as far as axon terminals to assist in local RNA translation [378–383]. Furthermore, TDP-43 has been found within the mitochondrial inner membrane and genetic analysis suggests it contains several mitochondrial targeting sequences [384–387].

Mitochondria are double-membraned organelles with their own DNA (mtDNA) encoding components of the oxidative phosphorylation pathway, regulated by a mix of nuclear transcription factors and mitochondrial-specific species [388, 389]. It is not unexpected to find TDP-43 within the mitochondrion; proper mitochondrial function depends on shuttling of transcripts from the nucleus and stabilization of transcripts during import and within the matrix. TDP-43’s amino acid sequence contains putative mitochondrial targeting sequences at aa35-41, aa146-150 and aa294-300, and may utilize proteins like peptidase mitochondrial processing alpha subunit (PMPCA) or Tom70 to facilitate mitochondrial entry, although this mechanism is still poorly understood [378, 385, 390–392]. Imbalances in TDP-43 can lead to significant mitochondrial dysfunction and oxidative stress [385–387, 393–400]. This may be through direct RNA regulation or through the regulation of other transcription factors present in the mitochondria, including MTFR1, a mitochondrial fission regulator [401–406].

Although typically treated as polycistronic and subject to self-splicing mechanisms, mtRNA may also be subject to cell-type-specific splicing events via transport of spliceosome-associated components from the nucleus into the mitochondria [389, 407]. Known transcriptional functions of TDP-43 include direct interactions with L-strand transcripts ND3, ND6, and a few mt-tRNAs, regulating production of complex I components based on mitochondrial levels of TDP-43 [385, 386]. Further work into TDP-43 transport or context-dependent binding could identify the mechanism by which TDP-43 loss leads to oxidative stress.

In short, spatial imbalance of TDP-43 could alter mitochondrial dynamics in several ways, including altered protein-protein interactions, altered protein-RNA interactions, and altered transport of species [331, 385, 399, 408]. Current evidence supports the existence of all of these functions as well as a role for TDP-43 as a modulator of mtRNA and mitochondrial-targeted mRNA stability in disease contexts, but more research is needed to better understand where TDP-43 has the largest impact.

### Viruses and TDP-43

Despite the focus on TDP-43 in the context of neuropathology, it was first discovered associated with the TAR regulatory element in HIV-1 [1]. By binding DNA transcribed from this region of the virus, TDP-43 was shown to regulate HIV-1 expression. Newer work suggests the virus can evade suppression by inducing caspase-3 cleavage of TDP-43 to TDP-35 [409], which then upregulate interferon (IFN) production through degradation of E3 ligase *RBCK1* pre-mRNA [410]. As *RBCK1* is also a cryptic exon-containing gene, it is possible that TDP-43 depletion in neurodegeneration may lead to a similar upregulation of the antiviral immune response. Furthermore, other viruses, such as SARS-CoV-2, have also been shown to induce cleavage and subsequent aggregation of TDP-43 [411–414]. Consistent with virus-induced TDP-43 dysregulation, cryptic exons in *UNC13A*, *ATG4B,* and *GPSM2* have been measured in HIV-1-infected SH-SY5Y cells [409].

Given the viral tendency to hijack cellular processes, it is unsurprising that TDP-43 has also been associated with other roles in the viral life cycle. The SARS-CoV-2 RNA-binding nucleocapsid protein may phase separate with and sequester RBPs, including TDP-43, when forming its virion [415, 416], while the influenza A virus may co-opt TDP-43 during production of viral mRNA [417]. Considering these examples, the observed neurodegeneration symptoms associated with HIV [418–420] and SARS-CoV-2 [421], and the suspected contributions of viral infections to neurodegeneration [422], the relationship between viruses and TDP-43 warrants further study.

### Other functions of TDP-43

While splicing regulation within the nucleus appears to be one of the main functions of TDP-43, it has also been implicated in a variety of other cellular processes and spaces. Based on CLIP-sequencing and mass spectrometry data, TDP-43 interacts with many RNAs and proteins, including, but not limited to, targets with compartment-specific enrichment. This implicates it in compartment-specific regulation, such as mitochondrial transcription [405, 406]. Other examples are also included within this section.

TDP-43 function and the formation of SGs and other MLOs are interlinked. While, as mentioned, TDP-43 localization can be altered because of cellular stress, appropriate SG formation also requires TDP-43. For example, *G3BP1*, a key gene in SG formation, is both stabilized by 3’UTR binding of TDP-43 to the mRNA transcript and subject to splicing-dependent regulation of a cryptic exon within the gene [54, 199, 281, 423, 424].

Advances in structural biology and microscopy have allowed the delineation of subcellular and subnuclear structures beyond the boundary of the nuclear envelope. Within the nucleus, TDP-43 can be found in nucleoli, paraspeckles, promyelocytic leukemia protein (PML) bodies, and Cajal bodies, and was even identified to be involved in centrosomal support [425]. Nucleolar inclusion of TDP-43 may be part of a stress response [426], but association with paraspeckles, Cajal bodies, and PML bodies are thought to be part of TDP-43’s normal function [427]. Cajal bodies are nuclear MLOs involved in modifying small nuclear ribonucleoproteins (snRNPs) for splicing, and TDP-43 likely plays a role in regulating the density and activity of these nuclear bodies [428, 429].

Additionally, TDP-43 suppresses transposons like LINE-1 and human endogenous retrovirus K (HERV-K), which are present in multiple copies throughout the human genome and, when aberrantly expressed, can lead to pathological symptoms [430–438]. Expression of HERV-K envelope protein, for example, can lead to motor neuron dysfunction [439, 440]. Some patients with ALS have detectable HERV-K transcript expression and generate antibodies against the HERV-K envelope protein, suggesting it can be successfully translated [441–444]. Some transposable elements are shown to increase their expression with age, potentially due to a loss of suppression [445–447]. Because TDP-43 function may also decline with age (see section “Changes in TDP-43 function with aging”), it could be informative to further explore whether TDP-43 loss of function precedes de-repression of transposons in clinical disease, considering transposable element dysregulation may overlap transcriptionally with TDP-43 loss in ALS [448].

TDP-43 is also thought to regulate the transcription of some genes and long noncoding RNAs (lncRNAs). For example, TDP-43 can be found localized to the outer shell of nuclear paraspeckles [449, 450], and has been shown to bind an APA site on the promoter sequence of long noncoding RNA (lncRNA) *NEAT1*, a major component of nuclear paraspeckles. This initiates transcript degradation and effectively suppresses paraspeckle formation [427, 451, 452]. In addition, TDP-43 binds to pre-miRNA transcripts and interacts with the Dicer complex in human cells [453]. A role in miRNA stability suggests TDP-43 may assist in removing unwanted mRNA from cytoplasm not only through facilitating active degradation [194, 453]. TDP-43 appears to play a crucial role in facilitating RNA turnover in the axon through direct binding to UG-rich motifs, and dysfunction of TDP-43 counterintuitively leads to accumulation, not loss, of a subset of axonal RNAs [454]. As an example, while disruption of TDP-43 leads to changes in expression of miR-27b-3p and miR-181c-5p, these miRNAs also may feed back to reduce the expression of TDP-43’s own mRNA, forming a cycle of autoregulation [455, 456].

R-loops are a type of RNA-DNA hybrid structure that can form during transcription and which, when incorrectly produced, can lead to significant DNA damage. TDP-43 appears to be an important protein in preventing the accumulation of R-loops and thus may protect the genome as well [446, 457–459]. Although its major role is in binding to RNA, TDP-43 also provides structural support for the critical XRCC4-DNA ligase 4 complex during non-homologous end joining mediated repair of double-strand breaks [460], and is important in the normal function of CHD2, a chromatin remodeling protein dysregulated in ALS-FTD [461].

Outside of the nucleus, TDP-43 has been found associated with the ER-mitochondrial interface and with structural proteins CHCHD10, VAPB, and PTPIP51 [379, 381, 384–387, 398, 462–465]. TDP-43 also associates with several RBPs in complexes shuttled along axons in motor neurons, which support and regulate local protein synthesis at the neuromuscular junction, a function which is critical due to the sheer distance between the nucleus and axon terminal of many motor neurons [378]. Axonal transport and neuromuscular junction function depends on TDP-43-mediated regulation of critical transport proteins like RAB4, sequestration of key RNAs within RNP complexes, and the disruption of RNA stability to facilitate turnover, where TDP-43 loss from the axon terminal leads to high levels of transcript accumulation [408, 454, 466]. Because the initiating factors that lead to TDP-43 loss from the nucleus in human disease are still poorly understood, understanding the normal contributions of TDP-43 outside the nucleus is necessary to effectively target this protein therapeutically.

## Pathological TDP-43 causes dysregulated RNA splicing

### Cryptic exon inclusion as a result of TDP-43 nuclear depletion or dysfunction

TDP-43 dysfunction and nuclear depletion lead to aberrant RNA splicing and regulatory phenotypes [13, 14]. Importantly, nuclear depletion is not required for these cytotoxic effects; inefficient binding at the RRM alone is sufficient [18]. The loss of RRM function or depletion of TDP-43 compromises RNA splicing control, resulting in aberrant splicing and the incorporation of CEs [13].

mRNA inclusion of CEs is deleterious to both the transcriptome and proteome. Most CEs do not encode in-frame peptides and, instead, lead to PTCs which can trigger nonsense-mediated decay of the mRNA transcript [13, 300, 467]. Inclusion of these CEs which encode PTCs, also known as “poison exons”, can reduce protein expression and are often associated with neurodevelopmental disorders or neurodegeneration [468–479]. Cassette or exon-extension exons which encode in-frame CEs are far rarer than out-of-frame exons [13, 53]. Although none have been fully characterized, it is possible for in-frame CEs to impact protein function through mechanisms such as disrupted domains, altered structure, and altered interactions [480–487]. Additionally, CEs can also lead to early polyadenylation events [205, 488, 489] or alternative splice sites [375, 376, 490, 491]. Finally, although the majority of CEs appear to be in the coding regions of genes, CEs can also be in the 5’ or 3’ UTRs of target genes. As these regions play a significant role in transcriptional regulation, alterations and aberrant splicing have the capability to significantly influence gene expression levels [492].

As a result, many of these mRNA-level changes due to TDP-43 dysregulation and CE inclusion impact the cellular transcriptome by reducing levels of mRNA encoding functional proteins [13, 274, 277–279, 335, 493–495]. This further impacts the proteome and results in decreased protein levels of the genes containing CEs [54, 285, 286, 488, 489, 496]. Furthermore, although CE identification is usually conducted in model systems of TDP-43 dysregulation and depletion, it is important to note that they are expressed at measurable levels in patient tissue and biofluids [22, 23, 30, 32, 33, 42, 53, 54, 285, 286, 488, 489, 497].

### Skiptic exons as a result of TDP-43 overaccumulation

While the nuclear depletion of TDP-43 generally leads to CE inclusion, models of TDP-43 overexpression display exon skipping events, which have been termed “skiptic” [498, 499]. TDP-43 overexpression, even of protein with mutant NLS, causes increased nuclear levels. As a result, the protein may bind lower affinity UG motifs near constitutive exons and induce exon repression. However, as a caveat, some skiptic events, such as that in *KCNQ2*, are known to result from TDP-43 depletion [32, 337, 500–502]. Better understanding the physiological roles of TDP-43 in transcription regulation could clarify whether non-overexpression-related skiptic events are due to a secondary role of the protein or due to a downstream effect of its loss. Still, CEs appear to be the dominant splicing phenotype stemming from nuclear depletion.

### Changes in TDP-43 function with aging

Aging is associated with the decline of many fundamental biological processes, including the accuracy of splicing machinery [503–507]. Splicing factors, including TDP-43, are found to be downregulated in aged models [508–510]. In a transdifferentiated aged neuron model, TDP-43 is mislocalized to the cytoplasm and exhibits decreased binding to cryptic target mRNA transcripts [511] and elderly patients without other neurodegenerative disorders present with TDP-43 pathology, including nuclear clearance [512–514]. In 2019, the term “limbic-predominant age-related TDP-43 encephalopathy” (LATE) was introduced to describe TDP-43 cytoplasmic aggregates in aged individuals as the principal pathology in the absence of other co-pathology, although comorbidity with Alzheimer’s disease and hippocampal sclerosis is common [21]. Since then, studies have shown that TDP-43 nuclear clearance and splicing abnormalities occur much earlier than aggregation, with cryptic targets detectable in individuals as young as their 50s, although seed amplification assays may be able to detect TDP-43 aggregates in CSF shortly before symptom onset in patients who go on to develop ALS-FTD [22, 23, 514–516]. Likewise, sporadic ALS iPSC-derived neurons subjected to aging exhibit evidence of TDP-43 loss of function as evidenced by presence of cryptic exons [517].

### TDP-43 dysfunction in human disease

#### Early-stage ALS-FTD involves nuclear clearance and splicing abnormalities

TDP-43 was first identified in a neurodegenerative context as a major component of neuronal and glial protein aggregates found in post-mortem tissue of ALS-FTD patients. Early studies focused on cytoplasmic aggregates in which TDP-43 was hyperubiquitinated, hyperphosphorylated, and cleaved such that a C-terminal fragment had been generated. While concurrent nuclear clearance was also observed, investigations centered on potential gain-of-function mechanisms through which the aggregates could cause disease [8, 11, 12].

Longitudinal studies of patient tissues and biofluids further indicate TDP-43 clearance occurs prior to the aggregation commonly observed in post-mortem tissue. As an example, a known FTD patient underwent brain resection to treat epilepsy prior to developing FTD-associated symptoms. Her pre-symptomatic brain tissue contained many neurons displaying nuclear clearance of TDP-43 but almost none with inclusions. However, post-symptomatic brain tissue was found to contain extensive aggregates, suggesting nuclear clearance preceded cytoplasmic aggregation [518]. In other cases, the protein product of the in-frame CE in *HDGFL2*, a target of TDP-43, has been measured in pre-symptomatic patient CSF and plasma [53], indicating TDP-43-associated RNA dysregulation occurs prior to symptomatic presentation of ALS-FTD. As a result, nuclear depletion of TDP-43 likely is an early-stage pathology and associated splicing deficits occur far earlier than originally expected.

**Fig. 3:**
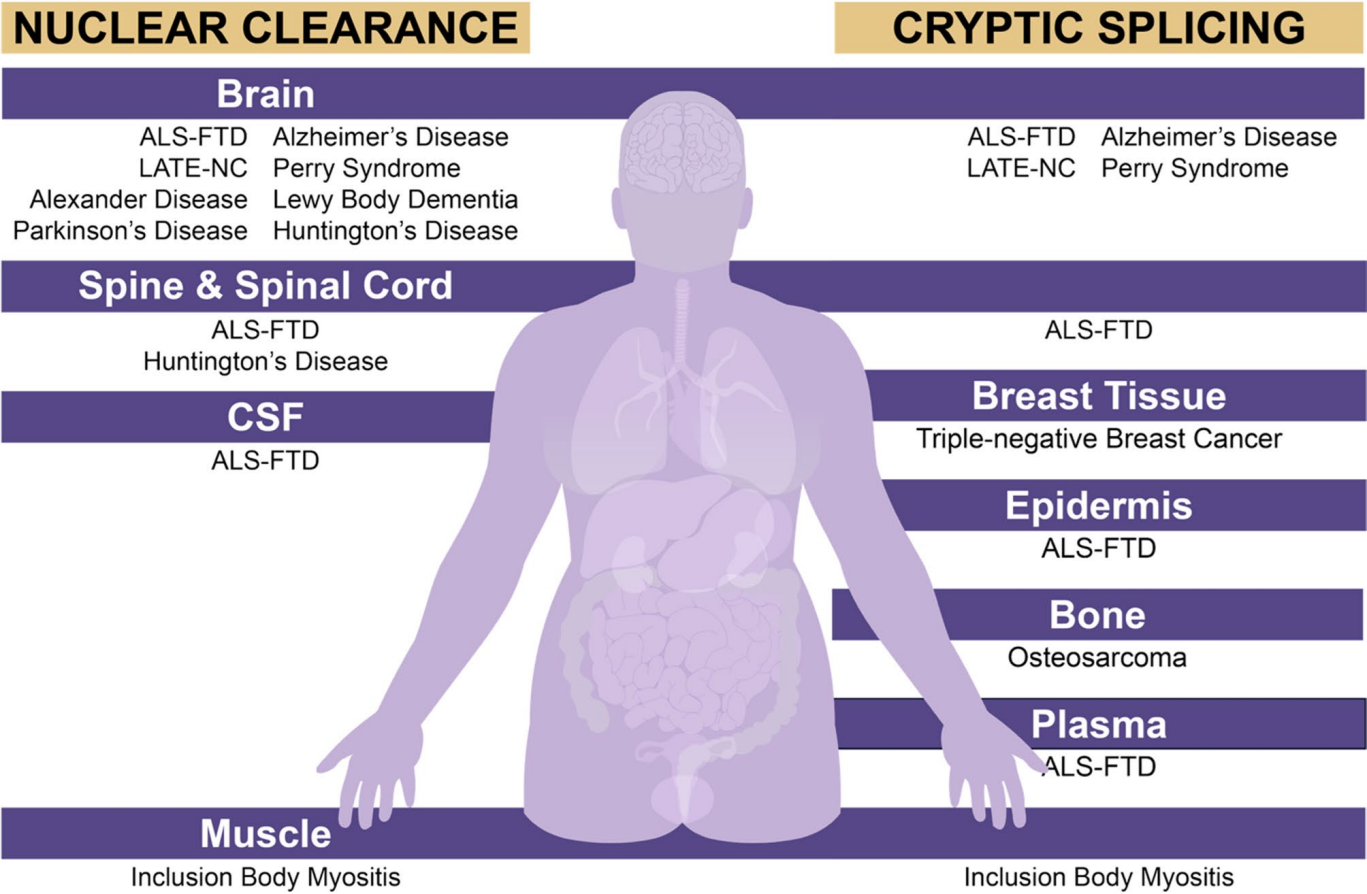
TDP-43 dysfunction detected in patient tissues. TDP-43 nuclear clearance and associated cryptic exons are measurable in human tissues. Summarized here and in and Tables 1, 2 are tissues and diseases in which the pathologies have been identified

ALS-FTD patients also exhibit TDP-43 splicing pathology in end-stage tissues (Fig. 3). Although complete TDP-43 loss can induce enough dysfunction to lead to cell death, surviving cells likely experience sustained splicing dysregulation, contributing to ongoing cellular stress. Cryptic exons have been detected at both RNA and protein levels in patient tissues, although measured targets are likely impacted by survivorship bias (Fig. 3) [32, 285, 286, 488, 489, 497, 515]. Many CEs likely contribute to protein dysfunction throughout the disease and impact proteins and pathways with important roles in neuronal health [20, 54, 366, 502, 554–557]. CEs in *STMN2* and *UNC13A* have been proposed as key contributors to disease and therapeutics designed to suppress them are currently being tested [287, 342, 558]. Others, such as the CE peptides in proteins such as HDGFL2 and MYO18A, have additionally been considered as biomarkers for TDP-43 nuclear depletion [53, 54, 559].

**Table 1:** Nuclear clearance of TDP-43 in human disease

Disease	Disease Subtype	Tissue/Region	Cell Types	References
Amyotrophic Lateral Sclerosis	N/A	Frontal Cortex	Neuron Glial cell	[Hasegawa 2009 pmid: 18546284] [140] [Gregory 2019 pmid: 31515300] [519] [Waldron 2025 PMID: 41256466] [514]
		Motor Cortex	Neuron Betz Cells	[Geser 2008 pmid: 18474740] [520] [Braak 2017 pmid: 27757524] [521] [Coyne 2021 pmid: 34321318] [522] [Du 2024 pmid: 39464100] [523] [Mackenzie 2007 pmid: 17469116] [524]
		Sensory Cortex	Neuron	[Geser 2008 pmid: 18474740] [520]
		Temporal Lobe/Insular Region	Neuron Glial cells Von Economo neuron Fork cells	[Geser 2008 pmid: 18474740] [520] [Spence 2024 pmid: 38443601] [525] [Gregory 2019 pmid: 31515300] [519] [Nana 2019 pmid: 30511086] [526]
		Cingulate Cortex	Oligodendrocyte Projection neuron Pyramidal cell Von Economo neuron	[Geser 2008 pmid: 18474740] [520] [Braak 2018 pmid: 29186496] [527]
		Hippocampus	Neuron	[Mackenzie 2007 pmid: 17469116] [524] [Waldron 2025 PMID: 41256466] [514]
		Amygdala	Neuron	[Waldron 2025 PMID: 41256466] [514]
		Neostriatum	Neuron	[Mori 2008 pmid: 18560845] [528]
		Substantia Nigra	Neuron	[Geser 2008 pmid: 18474740] [520]
		Brainstem	Neuron α-Motor Neuron	[Davidson 2007 pmid: 17219193] [12]
		Spine & Spinal Cord	Anterior Horn Cell Glial cell α-Motor Neuron	[Mori 2008 pmid: 18560845] [528] [Arai 2006 pmid: 17084815] [11] [Davidson 2007 pmid: 17219193] [12] [Braak 2017 pmid: 27757524] [521] [Trist 2022 pmid: 36008843] [529]
	I113T SOD1	Spinal Cord	Motor Neuron	[Trist 2022 pmid: 36008843] [529] [Robertson 2007 pmid: 17543992] [530]
	D101G SOD1	Spinal Cord	Motor Neuron	[Trist 2022 pmid: 36008843] [529] [Robertson 2007 pmid: 17543992] [530]
	C9ORF72 Repeat Expansion	Spinal Cord	Motor Neuron	[Trist 2022 pmid: 36008843] [529]
Frontotemporal Dementia (FTD)	Frontotemporal Dementia without MND	Frontal Lobe & Cortex	Neuron	[Neumann 2006 pmid: 17023659] [8] [Hatanpaa 2008 pmid: 18379440] [531] [Arseni 2022 pmid: 34880495] [167] [Vatsavayai 2016 pmid: 27797809] [518]
		Motor Cortex	Neuron	[Arseni 2022 pmid: 34880495] [167]
		Temporal lobe & Cortex	Neuron	[Kertesz 2016 pmid: 24479957] [532] [Vatsavayai 2016 pmid: 27797809] [518]
		Hippocampus	Neuron Granular Cells (Neuron)	[Higashi 2007 pmid: 17963732] [27] [Nakashima-Yasuda 2007 pmid: 17653732] [35] [Neumann 2006 pmid: 17023659] [8] [Kertesz 2016 pmid: 24479957] [532] [Hatanpaa 2008 pmid: 18379440] [531]
		Inferior Olivary Nucleus	Neuron	[Davidson 2009 pmid: 19330339] [533]
		Spine & Spinal Cord	Neuron	[Arseni 2022 pmid: 34880495] [167]
	Behavioral Variant FTD with VCP mutation p.Arg159His	Frontal Cortex	Neuron	[Sieben 2012 pmid: 22890575] [534]
		Temporal Cortex	Neuron	
	Primary Progressive Aphasia (PPA)	Amygdala	Neuron	[Bigio 2010 pmid: 20361198] [535]
	Progressive Supranuclear Palsy (PSP-S)	Dentate Gyrus	Neuron	[Yokota 2010 pmid: 20512649] [536]
		Entorhinal Cortex	Neuron	
	Corticobasal Syndrome (CBS)	Frontal Gyrus	Neuron	[Koga 2019 pmid: 29926172] [537]
		Thalamus	Neuron	
		Hypothalamus	Neuron	
		Subthalamic nucleus	Neuron	
		Brainstem	Neuron	
	FTD with Motor Neuron Disease (FTD-MD)	Frontal Cortex	Neuron	[Higashi 2007 pmid: 17963732] [27] [Ma 2022 pmid: 35197626] [286]
		Cerebral Cortex	Oligodendrocyte (TfR+)	[Higashi 2007 pmid: 17963732] [27]
		Temporal Cortex/Insular Region	Neuron Von Economo neuron Fork cells	[Hasegawa 2009 pmid: 18546284] [140] [Nana 2019 pmid: 30511086] [526]
		Hippocampus	Neuron	[Hasegawa 2009 pmid: 18546284] [140]
		Inferior Olivary Nucleus	Neuron	[Davidson 2009 pmid: 19330339] [533]
	Pick Disease	Temporal Cortex	Neuron	[Freeman 2008 pmid: 18091558] [538]
		Hippocampus	Neuron	
		Amygdala	Neuron	
Alzheimer’s disease (AD)	N/A	Frontal Cortex	Neuron	[Waldron 2025 PMID: 41256466] [514]
		Temporal Cortex	Neuron	[Higashi 2007 pmid: 17963732] [27]
		Hippocampus	Granule Neurons	[Nakashima-Yasuda 2007 pmid: 17653732] [35] [Sun 2017 pmid: 28332094] [30] [Waldron 2025 PMID: 41256466] [514] [Higashi 2007 pmid: 17963732] [27]
		Amygdala	Neuron	[Rita Agra Almeida Quadros, Li and Wang 2024 pmid: 38175301] [33] [Waldron 2025 PMID: 41256466] [514] [Higashi 2007 pmid: 17963732] [27]
		Inferior Olivary Nucleus	Neuron	[Davidson 2009 pmid: 19330339] [533]
	Limbic-predominant Age-related TDP-43 Encephalopathy neuropathologic change (LATE-NC)	Hippocampus	Neuron	[Chung 2024 pmid: 38308693] [22] [Chang 2023 pmid: 38133681] [435]
	Hippocampal Sclerosis (non-epileptic)	Cingulate Gyrus	Neuron	[Amador-Ortiz 2009 pmid: 17469117] [539]
		Hippocampus	Neuron	
		Amygdala	Neuron	
		Nucleus Accumbens	Neuron	
	AD + Dementia with Lewy Bodies (DLB)	Entorhinal Cortex	Neurons	[Nakashima-Yasuda 2007 pmid: 17653732] [35]
		Hippocampus	Neurons Granular neurons	[Nakashima-Yasuda 2007 pmid: 17653732] [35] [Higashi 2007 pmid: 17963732] [27]
Lewy Body Dementia (LBD)	N/A	Entorhinal Cortex	Neuron	[Nakashima-Yasuda 2007 pmid: 17653732] [35]
		Hippocampus	Granular cell neurons	
ALS/Parkinsonism Dementia	Brait-Fahn Schwartz Disease	Entorhinal Cortex	Neuron	[Hasegawa 2007 pmid: 17439983] [34]
		Hippocampus	Neuron	
Parkinson’s Disease (PD)		Hippocampus	Granular cell neurons	[Nakashima-Yasuda 2007 pmid: 17653732] [35]
	LRRK2 G2019S + MAPT variant	Substantia Nigra	Neuron	[Ling 2013 pmid: 23664753] [540]
	GRN mutation IVS0 + 5 G > C	Frontal Cortex	Neuron	[Sieben 2012 pmid: 22890575] [534]
		Temporal Cortex	Neuron	
Huntington’s Disease (HD)	Overlapping chorea with ALS	Motor Cortex	Pyramidal Neuron Glial Cell	[Tada 2012 pmid: 22735976] [541]
		Hippocampus (Dentate Gyrus)	Granular Neuron	
		Cervical Anterior Spinal Cord	Neuron	
	Multisystem Proteinopathy (fALS/Htt with VCP mutation)	Lumbar Anterior Spinal Cord	Neuron	[Oskarsson 2014 pmid: 25205077] [542]
Perry Syndrome	N/A	Substantia Nigra	Neuron	[Wider 2010 pmid: 18723384] [543]
Niemann-Pick C Disease	N/A	Cerebellum	Purkinje Cell	[Dardis 2016 pmid: 27193329] [544]
Alexander Disease	N/A	Frontal Lobe	Neuron	[Walker 2014 pmid: 24806671] [545]
Inclusion Body Myositis (IBM)	N/A	Muscle (skeletal, myofiber)	Muscle cell nuclei	[Salajegheh 2010 pmid: 19533646] [546] [Britson 2022 pmid: 35044790] [25] [Ikenaga 2025 pmid: 39757935] [26]
Multiple System Atrophy (MSA)	N/A	Motor Cortex	Neuron	[Nwabuobi 2019 pmid: 31745474] [547]
		Hippocampus	Neuron	[Koga 2018 pmid: 29660838] [548]
		Amygdala	Neuron	[Geser 2011 pmid: 20942898] [549] [Koga 2018 pmid: 29660838] [548]

Nuclear clearance of TDP-43 has been measured within various human tissues and cells

**Table 2:** TDP-43-associated cryptic exons in human disease

Disease	Brain Region/ Cell Type	CE Measured (non-exhaustive)	References
Amyotrophic Lateral Sclerosis	Prefrontal cortex	STMN2	[Grima 2025 PMID: 40275359] [550]
	Frontal Cortex	DLG5, EXD3, GOSR2, HDGFL2, IL18BP, KALRN, MARK3, MMAB, NDUFA5, POLDIP3, RAPGEF6, STMN2, STMN2, SYT, TBL1XR1-AS1, UNC13A, USP31	[Prudencio 2020 pmid: 32790644] [497] [Ma 2022 pmid: 35197626] [286] [Cao 2022 pmid: 35946434] [551] [Liu 2019 pmid: 31042469] [433] [Brown 2022 pmid: 35197628] [285] [Eshima 2023 PMID: 36609402] [552] [Sinha 2025 pmid: 40715064] [277] [Waldron 2025 PMID: 41256466] [514]
	Motor Cortex	AARS1, ADCY1, ADGRL1, AKT3, ATG4B, ATP8A2, ATXN1, BCL2L13, CAMK2B, CELF5, CLDN11, CORO7, CORO7-PAM16, CYFIP2, DOCK1, ELAVL3, EPB41L4A, GNG8, GPSM2, HDGFL2, HS6ST2, IGSF21, KALRN, KCNQ2, KIF23, KNCD1, MEIS2, MGAT5B, MYO9B, NCAPG2, PAOX, PHF2, POLDIP3, PRUNE2, SEPTIN7P2, SETD5, STMN2, SYNJ2, TMEM144, TMEM175, UNC13A, UNC13B, WASL, WDR35, ZNF195	[Grima 2025 PMID: 40275359] [550] [Prudencio 2020 pmid: 32790644] [497] [O’Neill 2025 PMID: 40067829] [448] [Shiga 2012 pmid: 22900096] [17] [Brown 2022 pmid: 35197628] [285] [Ling 2015 pmid: 26250685] [13] [Brown 2025 PMID: 41332610] [279]
	Temporal Cortex	ATG4B, GPSM2, STMN2	[Prudencio 2020 pmid: 32790644] [497] [Ling 2015 pmid: 26250685] [13]
	Hippocampus	STMN2	[Grima 2025 PMID: 40275359] [550] [Prudencio 2020 pmid: 32790644] [497]
	Thalamus	POLDIP3	[Shiga 2012 pmid: 22900096] [17]
	Occipital Cortex	STMN2	[Grima 2025 PMID: 40275359] [550]
	Cerebellum	STMN2	[Grima 2025 PMID: 40275359] [550]
	Spinal Cord	AARS1, AKT3, ARHGAP22, BACH1, BCL2L13, CDKAL1, CLDN11, DNM1, DOCK1, ELAVL3, EPB41L4A, GPSM2, IGFBP7–AS1, KCNQ2, KNCD1, PAOX, PHF2, POLDIP3, PRUNE2, RAB20, SEPTIN7P2, STMN2, STXBP5L, SYNJ2, UNC13B, WASL, ZNF382	[Klim 2019 pmid: 30643292] [488] [Melamed 2019 pmid: 30643298] [489] [O’Neill 2025 PMID: 40067829] [448] [Shiga 2012 pmid: 22900096] [17] [Alessandrini 2025 pmid: 41389796] [501] [Brown 2025 PMID: 41332610] [279]
	CSF	HDGFL2, KALRN, KCNQ2, MYO18A, RSF1, SYT7, SYNE1, CAMKIIB, SLC24A3, IGLON5, PXDN, SYN3, TRRAP	[Irwin 2024 pmid: 38278991] [53] [Seddighi 2024 pmid: 38277467] [54]
	Blood Plasma	HDGFL2	[Irwin 2024 pmid: 38278991] [53]
	Epidermis	STMN2	[Waldron 2025 PMID: 41256495] [514]
	Muscle	STMN2	[Waldron 2025 PMID: 41256495] [514]
Alzheimer’s disease	Entorhinal Cortex	STMN2, UNC13A	[Rita Agra Almeida Quadros, Li and Wang 2024 pmid: 38175301] [33]
	Hippocampus	ATG4B, CAMK2B, GPSM2, KCNQ2, STMN2, SYT7, UNC13A	[Sun 2017 pmid: 28332094] [30] [Ayuso 2023 pmid: 37605276] [32]
	Amygdala	ATG4B, CAMK2B, GPSM2, KCNQ2, STMN2, SYT7, UNC13A	[Sun 2017 pmid: 28332094] [30] [Ayuso 2023 pmid: 37605276] [23] [Rita Agra Almeida Quadros, Li and Wang 2024 pmid: 38175301] [33]
Frontotemporal Dementia	Frontal Cortex	AARS1, ACTL6B, ADCY1, ADCY7, ADGRL1, AKT3, ARHGAP22, ARHGAP32, ARL15, ATG4B, ATP8A2, ATXN1, C20orf194, CAMK2B, CDK7, CDO1, CELF5, CEP290, CEP72, CLDN11, CORO7, CORO7-PAM16, CYFIP2, DENND2B, DLG5, DNMT3A, DOCK1, EHD2RP4-583P15.15, ELAVL3, EPB41L4A, EXD3, FAM114A2, FGFR4, G2E3, GNB1L, GNG8, GOSR2, GPSM2, GRAMD1A, HAUS2, HDGFL2, HS6ST2, HS6ST3, IFT122, IGFBP7–AS1, IGSF21, IL18BP, INSR, IQCE, KALRN, KCNQ2, KIAA0753, KNCD1, LRP8, MAP7D2, MARK3, MEIS2, MGAT5B, MMAB, MTRR, MYO9B, NADSYN1, NCAPG2, NDUFA5, NPIPA3, NUP188, OSBP2, PAOX, PFKP, PHF2, PKN1, POLDIP3, PRELID3A, PRUNE2, PXDN, RAB20, RAP1GAP, RAPGEF6, RSF1, SEPTIN7P2, SERGEF, SETD5, STMN2, STXBP5L, SYNJ2, SYT7, TBL1XR1–AS1, TENM3, TMEM117, TMEM175, TRAPPC12, TRRAP, UBR5-DT, UNC13A, UPF2, USP31, WASL, WWOX, ZFP91, ZNF157, ZNF185, ZNF565, ZNF826P	[Ma 2022 pmid: 35197626] [286] [Cao 2022 pmid: 35946434] [551] [Liu 2019 pmid: 31042469] [433] [Brown 2022 pmid: 35197628] [285] [Prudencio 2020 pmid: 32790644] [497] [Brown 2025 PMID: 41332610] [279] [Belchikov 2025 PMID: 40913764] [553]
	Motor Cortex	ATG4B, GPSM2	[Ling 2015 pmid: 26250685] [13]
	Temporal Cortex	AARS1, ACTL6B, ADCY1, ADCY7, ADGRL1, AKT3, ARHGAP22, ARHGAP32, ARL15, ATG4B, ATP8A2, ATXN1, C20orf194, CAMK2B, CDK7, CDO1, CELF5, CEP290, CEP72, CLDN11, CORO7, CORO7-PAM16, CYFIP2, DENND2B, DNMT3A, DOCK1, EHD2RP4-583P15.15, ELAVL3, EPB41L4A, FAM114A2, FGFR4, G2E3, GNB1L, GNG8, GPSM2, GRAMD1A, HAUS2, HDGFL2, HS6ST2, HS6ST3, IFT122, IGFBP7-AS1, IGSF21, INSR, IQCE, KALRN, KCNQ2, KIAA0753, KNCD1, LRP8, MAP7D2, MEIS2, MGAT5B, MTRR, MYO9B, NADSYN1, NCAPG2, NPIPA3, NUP188, OSBP2, PAOX, PFKP, PHF2, PKN1, PRELID3A, PRUNE2, PXDN, RAB20, RAP1GAP, RSF1, SEPTIN7P2, SERGEF, STMN2, SETD5, STXBP5L, SYNJ2, SYT7, TENM3, TMEM117, TMEM175, TRAPPC12, TRRAP, UBR5-DT, UNC13A, UPF2, WASL, WWOX, ZFP91, ZNF157, ZNF185, ZNF565, ZNF826P	[Prudencio 2020 pmid: 32790644] [497] [Brown 2025 PMID: 41332610] [279] [Ling 2015 pmid: 26250685] [13]
	Amygdala	CAMK2B, KCNQ2, STMN2, SYT7, UNC13A	[Ayuso 2023 pmid: 37605276] [23]
	Hippocampus	CAMK2B, KCNQ2, STMN2, SYT7, UNC13A	[Ayuso 2023 pmid: 37605276] [23]
	Basal Ganglia (Caudate Nucleus)	ACTL6B, ATG4B, CAMK2B, GPSM2, HDGFL2, KCNQ2, STMN2, UNC13A	[Pickles 2025 PMID: 39788898] [42]
	Substantia Nigra	ATG4B, GPSM2, STMN2, UNC13A	[Pickles 2025 PMID: 39788898] [42]
	Blood Plasma	HDGFL2	[Irwin 2024 pmid: 38278991] [53]
	CSF	CAMKIIB, HDGFL2, IGLON5, KALRN, KCNQ2, MYO18A, PXDN, RSF1, SLC24A3, SYN3, SYNE1, SYT7, TRRAP	[Irwin 2024 pmid: 38278991] [53] [Seddighi 2024 pmid: 38277467] [54]
Limbic-predominant Age-related TDP-43 Encephalopathy (LATE)	Amygdala	HDGFL2	[Chang 2023 pmid: 38133681] [435]
	Hippocampus	ARHGAP32, CAMK2B, ELAVL3, HDGFL2, KALRN, PFKP, STMN2, SYT7, UNC13A	[Chung 2024 pmid: 38308693] [22] [Chang 2023 pmid: 38133681] [435] [Trautwig 2025 PMID: 40501554] [55]
Perry Syndrome	Basal Ganglia (Caudate Nucleus)	ACTL6B, ATG4B, CAMK2B, GPSM2, HDGFL2, KCNQ2, STMN2, UNC13A	[Pickles 2025 PMID: 39788898] [42]
	Substantia Nigra	UNC13A	[Pickles 2025 PMID: 39788898] [42]
Inclusion Body Myositis (IBM)	Skeletal Muscle	ACSF2, GPSM2, HDGFL2, ZFP91	[Britson 2022 pmid: 35044790] [25] [Ikenaga 2025 pmid: 39757935] [26]
Osteosarcoma	Bone	HDGFL2	[Sinha 2025 pmid: 40715064] [277]
Triple Negative Breast Cancer	Breast Tissue	HDGFL2	[Sinha 2025 pmid: 40715064] [277]

Cryptic exons were measured at an mRNA level in all studies except for Irwin et al. 2024 and Seddighi et al. 2024, which detect the cryptic exons at a protein level

#### TDP-43 splicing dysfunction in other diseases

Beyond ALS-FTD and LATE, TDP-43 pathology is most extensively studied in Alzheimer’s disease (AD). A rare NLS mutation in *TARDBP* is associated with AD (Fig. 1B) and, although LATE can occur independently of AD, the two often co-occur [23, 31, 195]. Furthermore, cryptic *HDGFL2* and other CEs are measurable in AD tissue, both at the RNA and protein level, even in the absence of TDP-43 inclusions (Fig. 3) [23, 30, 33]. Outside of the nervous system, TDP-43 dysregulation and splicing deficits have also been measured in Inclusion Body Myositis (IBM), a myopathy [26]. Furthermore, the identification of TDP-43 dysfunction in some types of cancers has sparked some exploration into the contribution of TDP-43’s RNA-binding functions to malignancy, and potential points of interference include TDP-43 splicing of tumor associated factors, regulation of tumor-associated miRNAs, and repression of lncRNAs [560].

CEs and other transcriptomic alterations remain underexplored in many conditions associated with TDP-43 pathology, as research has traditionally focused on protein aggregation as the primary pathogenic mechanism. Nuclear clearance in end-stage tissue suggests these other conditions may also involve early-stage splicing abnormalities associated with TDP-43 depletion (Table 1) [4, 23, 25–27, 30, 33–35, 514, 533, 534, 539–549]. However, as recognition of CEs grows, reports of their detection across a wider range of disorders are becoming more common (Fig. 3, Table 2) [13, 17, 22, 23, 25, 26, 30, 32, 33, 42, 53–55, 277, 279, 285, 286, 424, 433, 448, 488, 489, 497, 501, 514, 550–553].

#### End-stage disease tissues display TDP-43 aggregation in varied disorders

Although this review focuses on the loss-of-function mechanisms of TDP-43 in disease, its aggregation pathology was recognized first and is highly characterized. Upwards of 90% of ALS and 50% of FTD cases have been found to display insoluble TDP-43 aggregates [196, 561, 562]. Cytoplasmic aggregation of TDP-43 has been detected in many disorders, including AD [27, 28, 539, 563–565], Huntington’s disease (HD) [566], Parkinson’s disease (PD) [35, 567], and others [4, 34, 545, 548, 568–575].

Ubiquitin-positive tau-negative TDP-43 inclusions were first described within the ALS-FTD disease spectrum [8, 11, 12]. Since then, these cytoplasmic inclusions have been found within both neurons and glia across many diseases, including ALS-FTD, IBM, and Alzheimer’s disease. In addition to ubiquitination, TDP-43 aggregates found in ALS-FTD are also commonly truncated and hyperphosphorylated [2, 8, 11, 12, 141]. When extracted, these inclusions are sarkosyl-insoluble and are able to seed the aggregation of TDP-43 fragments in new contexts. Staging of TDP-43 pathology in ALS-FTD and AD reveals distinct patterns through which the inclusions spread over time [28, 576]. Based on the pattern of early-stage nuclear clearance and dysfunction, followed eventually by end-stage aggregation in ALS-FTD and AD, similar trends may underlie other diseases exhibiting TDP-43 aggregation.

The exact cause of TDP-43 aggregation remains unknown and aggregates may take different forms in different diseases and contexts, with its formation tendencies impacted by factors such as *TARDBP* mutations [169, 190, 194, 202, 577] and the presence of other pathological proteins (PMIDs): 17963732 [27], 39596445 [271], 29134321 [578], 38212334 [169], 37037195 [579], 38052886 [580], 33771571 [581], 33230138 [582], 33065281 [583], 30837838 [194], 29660838 [548], 27545621 [190], 27694152 [584], 24591609 [202], 21454607 [577], 21376022 [585], 19018245 [566]. Some studies also implicate intra-condensate de-mixing as a result of multiple stressors as a key step for TDP-43 aggregation [156]. Although TDP-43 aggregates are commonly found within end-stage tissues, reduction of aggregation in gain-of-function models has been unable to consistently reduce cell death [586]. However, aggregation of the protein is itself a suppressor of its normal function [587]. To this end, some studies have explored aggregation inhibition [194, 588–592] while others have looked at the efficacy of molecular chaperones and disaggregases in renewing protein function through reversing aggregation and restoring nuclear localization [593–600].

## Discussion

Accurate splicing of pre-mRNA is a process crucial for maintaining the cellular transcriptome and, thus, proteome. Alternative splicing of human genes is often context-specific and can lead to phenotypic differences [601–608]. Splicing fidelity is maintained, in part, by RNA-binding proteins (RBPs) which associate with pre-mRNA transcripts and influence splice site selection. Three large families of these proteins are heterogeneous nuclear RNPs (hnRNPs), which include PTBP1 and TDP-43, ELAV/Hu, and serine-arginine-rich (SR) proteins [609–617]. These proteins have varied structures of RNA-binding domains and target different RNA motifs. Often, these proteins will act in concert to effectively guide splicing [603, 618].

Over the past few decades, TDP-43 has evolved from a little-known protein to one recognized as a key regulator of RNA with significant implications in diseases in the CNS, such as ALS-FTD, to peripheral disorders, such as IBM. Although primarily localized to the nucleus, its effects span the entire cell. As an RNA-binding protein, TDP-43 acts to shuttle, stabilize, sequester, and direct splicing and expression. Its many known functions suggest it to be a vital piece in the complex interactions that regulate RNA of every kind, and raise further questions about its mechanisms of action and possible other functions the field has yet to uncover.

The rise of modern transcriptomics has led to the identification of splicing dysregulation as a common pathology found across neurodegenerative disorders [619]. Combined with the broadly observed nuclear depletion and aggregation of TDP-43, as well as its role in RNA splicing regulation, recent work suggests that TDP-43 plays a pivotal role in many neurodegenerative disorders. As a result, therapeutics and biomarkers targeting TDP-43 availability and restorating the transcriptome it regulates suggest a promising path forward in treating these disorders.

Current therapeutic strategies being researched to reverse the impacts of TDP-43 splicing dysfunction include antisense oligonucleotides (ASOs), modified U7 small nuclear RNAs (U7smOPT snRNAs), and transgene systems meant to express a modified and non-aggregating form of TDP-43. ASOs are short oligonucleotides designed to bind pre-mRNA near or at specific splice sites, thereby blocking the inclusion of these cryptic sequences in mature mRNA [620–626]. As a result, aberrant splicing and protein isoform translation are prevented and gene functionality will be restored. Currently, an ASO targeting the cryptic exon in STMN2 is being tested clinically and others, including ones targeting cryptic *UNC13A*, are in development [20, 342, 488, 554, 627–629]. While ASO technologies are TDP-43-level agnostic, a newer technology utilizing U7mOPT and transgene systems are meant to be more responsive to protein levels. A U7smOPT snRNA can be directed to specific cryptic exons using custom antisense sequences. Then, the complex will recruit hnRNP A1, a protein with a well-established role in RNA regulation [630, 631], to conduct TDP-43’s splicing function [632–635]. Directing snRNAs to TDP-43 binding sites should restrict splicing modulation to mRNA without bound TDP-43 and this method is capable of targeting multiple sites at once. However, this binary recognition may be ineffective at sites which require multivalent recognition or are only partially bound. Two novel transgene systems that regulate expression based on endogenous TDP-43 may be more efficacious in those cases. These systems have been used to express a TDP-43/RAVER1 fusion protein, in which the C-terminal aggregation-prone domain of TDP-43 has been replaced with the splicing repressor domain of RAVER1 [13, 333, 636–640]. When expressed at a physiologic level, this fusion protein rescues TDP-43-associated cryptic splicing, broadly correcting transcriptome-wide dysregulation. One approach fuses the 3’UTR autoregulatory domain of TDP-43 to the payload itself [13, 333, 360, 361]. Consequently, transgene mRNA expression is restricted to TDP-43-depleted cells and fusion protein production is tightly regulated by endogenous TDP-43 levels. Another approach uses “TDP-REG” vectors, which restrict transgene expression through a regulatory element containing *in silico-*engineered cryptic splicing events [558]. This design ensures the transgene is expressed only in cells where cryptic splicing, indicative of TDP-43 functionality, is triggered within the regulatory element.

The ubiquitous nature of TDP-43, non-specificity of many commercial antibodies, and its cell-type specific depletion make it a challenging target as a biomarker. Measurement of the protein itself with patient biofluids has proven difficult and often focuses on truncated or PTM-including forms of the protein. Recently developed PET ligand [^18^F]ACI-19626 may be able to detect TDP-43 aggregates, which could assist in late-stage diagnosis of TDP-43 proteinopathy [641]. Protein detection from blood is ideal for biomarker development, because a blood draw is far less invasive and expensive than other techniques. Many studies have explored the utility of identifying TDP-43 levels in blood through various methods, but a major challenge remains in identifying a consistent biomarker which differentiates TDP-43 proteinopathy from non-TDP-43 related disease [44–47, 49–52, 642–648].

A second emerging route towards distinguishing TDP-43 proteinopathies is the direct evaluation of cryptic peptides, the proteins produced through successful translation of cryptic mRNA. Although the majority of TDP-43-associated are degraded through nonsense mediated decay (NMD), approximately 3% encode in-frame peptides which do not trigger NMD [13, 53, 274, 277–279, 334, 649]. Additionally, some NMD does not act on all CE-containing transcripts with 100% efficiency, so some cryptic transcripts successfully produce protein products containing cryptic peptide sequences detectable at a protein level, even in presymptomatic patient biofluids [54]. The detection of cryptic proteins in patient biofluids offers a particularly promising approach because it allows researchers to understand the levels of TDP-43 function at an early stage of disease. TDP-43 depletion in peripheral tissues of patients may also enable other patient samples, such as skin, to be tested in the future [515]. Detection of cryptic HDGFL2 has since undergone exploration as a potential biomarker for IBM as well [25, 26, 56]. Current ELISA-based detection methods for proteins allow for scalability and require small amounts of sample. New NGS-based proteomics technologies may offer further benefits as the technology develops [650–652]. Combined with the potential for cryptic peptides to offer specificity in identifying TDP-43 proteinopathies, future multiplexed cryptic peptide assays may prove to be a highly sensitive, adaptable method for disease diagnostics and prognostics.

## Conclusions

In this review, we provide a broad overview of TDP-43 structure, biological functions, and pathology through the lens of its role in splicing regulation. Improvements in RNA-seq technologies have advanced our understanding of TDP-43 pathological mechanisms, including the identification of cryptic exons within mRNA. The ubiquitous nature of TDP-43, along with the increasing recognition of its pathology across disorders emphasize the need for a better understanding of the impacts of TDP-43 dysfunction. We highlight the connections between loss of function and key genetic and protein modifications, along with important regulatory mechanisms controlling TDP-43. We further investigate the presymptomatic presence of TDP-43 nuclear depletion and cryptic exons, and curate a collection of cases in which depletion and/or cryptic exons have been measured across different disorders (Tables 1, 2). However, the mechanisms behind TDP-43 nuclear depletion and dysfunction, along with downstream impacts across different contexts remain an active area of research. To this end, we have also curated datasets in which TDP-43 has been depleted in a rodent or human model (Table 3) to support future research and comparative analyses. Through synthesizing existing knowledge of TDP-43 splicing regulation, this study provides a resource for applications leveraging cryptic exons to detect, diagnose, and treat TDP-43 dysfunction occurring in major neurodegenerative disorders.

**Table 3:** Publicly available bulk RNA-Seq datasets from human, mouse, and rat models of TDP-43 depletion

Sample Characteristics				Library Prep		Reference		
Organism	Sample Source/Model	Cell Type	Method of Depletion	Selection	Instrument	BioProject	GEO	Reference
*Homo sapiens*	FTLD-TDP Middle frontal neocortex	Neuron nuclei	FACS with C-terminal anti-TDP43 antibody	cDNA	Illumina HiSeq 2500	PRJNA522295	GSE126543	[Liu 2019 PMID: 31042469] [433]
*Homo sapiens*	ALS Middle temporal gyrus	Pericytes (culture)	siRNA	polyA	Illumina NovaSeq 6000	PRJNA928099	GSE223747	[Cao 2023 PMID: 37527763] [334]
*Homo sapiens*	ALS Motor cortex	Pericytes (culture)	siRNA	polyA	Illumina NovaSeq 6000	PRJNA928099	GSE223747	[Cao 2023 PMID: 37527763] [334]
*Homo sapiens*	Umbilical cord endothelium	HUVEC	shRNA	polyA	Illumina HiSeq 2000	PRJNA976705	GSE233588	[Hipke 2023 PMID: 37384248] [653]
*Homo sapiens*	CV-hiPSC-B (hiPSC)	Motor neurons	shRNA	rRNA depletion & polyA	Illumina HiSeq 2000	SRP069787	GSE77702	[Kapeli 2016 PMID: 27378374] [654]
*Homo sapiens*	HUES3 (hESC)	Motor neurons	siRNA	polyA	Illumina HiSeq 2000	PRJNA497804	GSE121569	[Klim 2019 PMID: 30643292] [488]
*Homo sapiens*	771-3 G (hiPSC)	Motor neurons	shRNA	polyA	Illumina NextSeq 1000	PRJDB19918	N/A	[Tanaka 2025 PMID: 40670663] [655]
*Homo sapiens*	771-3 G (hiPSC)	Motor neurons	shRNA	polyA	ONT GridION	PRJDB19918	N/A	[Tanaka 2025 PMID: 40670663] [655]
*Homo sapiens*	CS83iCTR33-n1 with Htt 18Q or 50Q (hiPSC)	Medium Spiny Neurons	siRNA	rRNA depletion & polyA	Illumina NovaSeq 6000	PRJNA1166871	GSE278354	[Nguyen 2025 PMID: 39762660] [43]
*Homo sapiens*	WTC11 (hiPSC)	i^3^Neuron (cortical-like)	CRISPRi	polyA	Illumina HiSeq 2000	PRJEB42763	N/A	[Brown 2022 PMID: 35197628] [285]
*Homo sapiens*	WTC11 (hiPSC)	i^3^Neuron (cortical-like)	CRISPRi	Neurite vs. Soma; polyA	Illumina NovaSeq 6000	PRJNA1273942	GSE299341	[Dargan 2025 PMID: 40737092] [656]
*Homo sapiens*	WTC11 (hiPSC)	i^3^Neuron (cortical-like)	CRISPRi	polyA	Illumina HiSeq 2500	PRJNA1256902	GSE296714	[Bryce-Smith 2025 PMID: 41120751] [375]
*Homo sapiens*	WTC11 (hiPSC)	i^3^Neuron (cortical-like)	CRISPRi	polyA	Illumina NextSeq 500	PRJNA1235234	GSE307054	[Sinha 2025 PMID: 40667039] [277]
*Homo sapiens*	WTC11 (hiPSC)	i^3^Neuron (cortical-like)	CRISPRi	polyA	Unknown	Not yet available		[Kozareva 2025 PMID: 41256508] [496]
*Homo sapiens*	Immortalized cell line	BE (2)-M17	shRNA	polyA	DNBSEQ-T7	PRJNA1083198	GSE260737	[Gu 2025 PMID: 40202498] [657]
*Homo sapiens*	Immortalized cell line	SH-SY5Y	siRNA	polyA	Illumina HiSeq 2500	PRJNA381144	GSE97262	[Appocher 2017 PMID: 28575377] [262]
*Homo sapiens*	Immortalized cell line	SH-SY5Y	siRNA	polyA	Illumina HiSeq 4000	PRJNA503396	GSE122069	[Melamed 2019 PMID: 30643298] [489]
*Homo sapiens*	Immortalized cell line	SH-SY5Y	siRNA	rRNA depletion & polyA	Illumina NovaSeq 6000	PRJNA682459	GSE162644	[Dunker 2021 PMID: 33852834] [658]
*Homo sapiens*	Immortalized cell line	SH-SY5Y	shRNA	polyA	Illumina NovaSeq 6000	PRJEB42763	N/A	[Brown 2022 PMID: 35197628] [285]
*Homo sapiens*	Immortalized cell line	SH-SY5Y	shRNA	polyA	Illumina HiSeq 2500	PRJNA961085	GSE230408	[Hou 2024 PMID: 38184854] [446]
*Homo sapiens*	Immortalized cell line	SH-SY5Y	shRNA	polyA	Illumina HiSeq 2500	PRJNA1256902	GSE296713	[Bryce-Smith 2025 PMID: 41120751] [375]
*Homo sapiens*	Immortalized cell line	SH-SY5Y	CRISPRi	polyA	Illumina HiSeq 2500	PRJNA1238221	GSE292352	[Watanabe 2025 PMID: 40707625] [659]
*Homo sapiens*	Immortalized cell line	SK-N-BE(2)	shRNA	polyA	Illumina HiSeq 2500	PRJNA1256902	GSE296711	[Bryce-Smith 2025 PMID: 41120751] [375]
*Homo sapiens*	Immortalized cell line)	SK-N-DZ	siRNA	polyA	9 Illumina NovaSeq 6000	PRJEB42763	N/A	[Brown 2022 PMID: 35197628] [285]
*Homo sapiens*	Immortalized cell line)	HeLa M	CRISPR/Cas9	polyA	Illumina HiSeq 2500	PRJNA562297	GSE136366	[Roczniak-Ferguson 2019 PMID: 31527135] [660]
*Homo sapiens*	Immortalized cell line)	HeLa	siRNA	polyA	Illumina HiSeq 2000	PRJNA282692	N/A	[Ling 2015 PMID: 26250685] [13]
*Mus musculus*	C57Bl/6J Spleen & Lymph node	CD8+ T cells	CRISPR/Cas9	polyA	Illumina NovaSeq 6000	PRJNA823722	GSE200238	[Karginov 2022 PMID: 35725727] [661]
*Mus musculus*	B6D2F1 Embryo	Mixture	siRNA	rRNA depletion & cDNA	Illumina HiSeq X Ten	PRJNA818863	GSE199197	[Li 2022 PMID: 36417507] [434]
*Mus musculus*	Zygote	Mixture	Zp3-Cre; Tardbp f/f	polyA	Illumina NovaSeq 6000	PRJNA917193	GSE221985	[Nie 2023 PMID: 37460529] [662]
*Mus musculus*	Metaphase II oocyte	Mixture	Zp3-Cre; Tardbp f/f	polyA	Illumina NovaSeq 6000	PRJNA917193	GSE221985	[Nie 2023 PMID: 37460529] [662]
*Mus musculus*	Early 2-cell embryo	Mixture	Zp3-Cre; Tardbp f/f	polyA	Illumina NovaSeq 6000	PRJNA917193	GSE221985	[Nie 2023 PMID: 37460529] [662]
*Mus musculus*	Late 2-cell embryo	Mixture	Zp3-Cre; Tardbp f/f	polyA	Illumina NovaSeq 6000	PRJNA917193	GSE221985	[Nie 2023 PMID: 37460529] [662]
*Mus musculus*	Full-grown oocyte	Mixture	Zp3-Cre; Tardbp f/f	polyA	Illumina NovaSeq 6000	PRJNA917193	GSE221985	[Nie 2023 PMID: 37460529] [662]
*Mus musculus*	Cortex	Mixture	Nestin-Cre; Tardbp f/f	polyA	Illumina HiSeq 4000	PRJNA1217796	GSE288457	[Agrawal 2025] [663]
*Mus musculus*	Intestine	Crypt cells	Villin-Cre^ERT2^; Tardbp f/f	polyA	Illumina HiSeq 2500	PRJNA945464	GSE227532	[Yang 2023 PMID: 38157451] [477]
*Mus musculus*	Mammary gland	Mixture	WAP-Cre; Tardbp f/f	polyA	Illumina HiSeq 2000	PRJNA478555	GSE116456	[Zhao 2020 PMID: 31953403] [664]
*Mus musculus*	Spinal cord	Embryonic stem (ES) cell	Cre^ERT^; Tardbp f/f	polyA	Illumina Genome Analyzer	PRJNA127211	GSE21993	[Chiang 2010 PMID: 20660762] [63]
*Mus musculus*	Spinal cord	Mixture	Gfap-Cre; Tardbp f/f	polyA	Illumina HiSeq 4000	PRJNA658318	GSE156542	[Peng 2020 PMID: 33127758] [665]
*Mus musculus*	CD-1 Spinal cord	Motor neuron (culture)	Somatodendritic & Axonal; polyA	shRNA	Illumina MiSeq	PRJNA615680	GSE147607	[Briese 2020 PMID: 32709255] [666]
*Mus musculus*	Spinal cord	Endothelial	Cdh5(PAC)-Cre^ERT2^; Tardbp f/f	polyA	Illumina NovaSeq 6000	PRJNA1067587	GSE253868	[Arribas 2024 PMID: 38300714] [667]
*Mus musculus*	Sciatic nerves	Mixture	Cnp-Cre; Tardbp f/f	polyA	Illumina HiSeq 4000	PRJNA662527	GSE157714	[Chang 2021 PMID: 33689679] [668]
*Mus musculus*	C57Bl/6J Striatum	Mixture	ASO	polyA	Illumina Genome Analyzer II	PRJNA141971	GSE27218	[Polymenidou 2011 PMID: 21358643] [14]
*Mus musculus*	C57Bl/6J Striatum	Mixture	ASO	polyA	Illumina NovaSeq 6000	PRJNA1166871	GSE278354	[Nguyen 2025 PMID: 39762660] [43]
*Mus musculus*	Testis	Mixture	Stra8-Cre; Tardbp f/f	polyA	Illumina NovaSeq 6000	PRJNA733459	GSE175734	[Campbell 2021 PMID: 34599968] [669]
*Mus musculus*	Thalamus	Mixture	Nestin-Cre; Tardbp f/f	polyA	Illumina HiSeq 4000	PRJNA1217796	GSE288457	[Agrawal 2025] [663]
*Rattus norvegicus*	Neonatal cortex	Astrocytes	siRNA	polyA	Illumina HiSeq 2000	PRJNA559433	GSE135611	[LaRocca 2019 PMID: 31229690] [670]

All datasets include samples with known TDP-43 depletion

## Methods

### Identification of cases with TDP-43 nuclear clearance in human disease

The following searches were conducted in between 7/10/2025 and 12/18/2025 to identify examples of TDP-43 nuclear clearance in human disease conditions:

Search 1: (tdp-43[All Fields] AND nuclear[All Fields] AND pathology[All Fields])

Search 2: (tdp-43[All Fields] AND nuclear[All Fields] AND clearance{All Fields])

Search 3: (nuclear[All Fields] AND tdp-43[All Fields] AND ALS[All Fields])

Search 4: (nuclear[All Fields] AND tdp-43[All Fields] AND Alzheimer’s Disease[All Fields])

Search 5: (nuclear[All Fields] AND tdp-43[All Fields] AND Frontotemporal Dementia[All Fields])

### Identification of cases with TDP-43-associated cryptic exons in human disease

The following searches were conducted in between 7/25/2025 and 12/18/2025 to identify examples of TDP-43 cryptic exons in human disease conditions:

Search 1: (tdp-43[All Fields] AND nuclear[All Fields] AND pathology[All Fields])

Search 2: (tdp-43[All Fields] AND cryptic exon expression[All Fields])

Search 3: (tdp-43[All Fields] AND splicing[All Fields] AND pathology[All Fields])

Search 4: (nuclear[All Fields] AND tdp-43[All Fields] AND ALS[All Fields])

Search 5: (nuclear[All Fields] AND tdp-43[All Fields] AND Alzheimer’s Disease[All Fields])

Search 6: (nuclear[All Fields] AND tdp-43[All Fields] AND Frontotemporal Dementia[All Fields])

### Identification of RNA-Seq datasets investigating TDP-43 depletion phenotypes in human and rodent contexts

The following searches were conducted in between 8/1/2025 and 12/18/2025 to identify RNA-Seq datasets modeling TDP-43 depletion in human and rodent contexts:

On GEO:

Search 1: (tdp-43[All Fields] AND knockout[All Fields]) AND (“gse”[Filter] AND “Expression profiling by high throughput sequencing”[Filter])

Search 2: (“tdp-43 KO”[All Fields]) AND (“gse”[Filter] AND “Expression profiling by high throughput sequencing”[Filter])

Search 3: “tdp-43 knockdown”[All Fields] AND (“gse”[Filter] AND “Expression profiling by high throughput sequencing”[Filter])

Search 4: “tdp-43 knockdown”[All Fields] AND (“gse”[Filter] AND “Expression profiling by high throughput sequencing”[Filter])

Date: 8/6/25

Search 5: (“tardbp”[All Fields] AND “knockout”[All Fields]) AND (“gse”[Filter] AND “Expression profiling by high throughput sequencing”[Filter])

On BioProject:

Search 1: (TDP-43[All Fields] AND knockdown[All Fields]) AND “bioproject sra”[Filter] AND “method sequencing”[Filter])

Search 2: (TDP-43[All Fields] AND knockout[All Fields]) AND (“bioproject sra”[Filter] AND “method sequencing”[Filter])

Search 3: (“tdp-43 KO”[All Fields]) AND (“transcriptome gene expression”[Filter] AND “bioproject sra”[Filter] AND “method sequencing”[Filter])

Search 4: (“tdp-43KO”[All Fields]) AND (“transcriptome gene expression”[Filter] AND “bioproject sra”[Filter] AND “method sequencing”[Filter])

Search 3: (“tdp-43 KD”[All Fields]) AND (“transcriptome gene expression”[Filter] AND “bioproject sra”[Filter] AND “method sequencing”[Filter])

Search 5: (“tdp-43KD”[All Fields]) AND (“transcriptome gene expression”[Filter] AND “bioproject sra”[Filter] AND “method sequencing”[Filter]

As identifying splicing abnormalities require full transcript alignments, scRNA-seq and array-based methods were excluded.

## Data Availability

Data sharing is not applicable as no datasets were generated or analyzed in the article.

## Abbreviations

AD: Alzheimer’s disease
ALS: Amyotrophic lateral sclerosis
APA: Alternative polyadenylation
ASO: Antisense oligonucleotides
CE: Cryptic exon
CLIP: Cross-linking and immunoprecipitation
CNS: Central nervous system
Cryo-EM: Cryogenic electron microscopy
CSF: Cerebrospinal fluid
DNA: Deoxyribonucleic acid
ELISA: Enzyme-linked immunosorbent assay
ER: Endoplasmic reticulum
FTD: Frontotemporal dementia
GWAS: Genome-wide association study
HERV-K: Human endogenous retrovirus K
HIV: Human immunodeficiency virus
hnRNP: Heterogeneous nuclear ribonucleoprotein
IBM: Inclusion body myositis
IDR: Intrinsically disordered region
IFN: Interferon
iPSC: Induced pluripotent stem cell
LATE: Limbic-predominant age-related TDP-43 encephalopathy
lncRNA: Long noncoding RNA
LINE-1: Long interspersed nuclear element-1
LLPS: Liquid-liquid phase separation
miRNA: MicroRNA
MLO: Membraneless organelle
mRNA: Messenger RNA
mtDNA: Mitochondrial DNA
mt-tRNA: Mitochondrial tRNA
NGS: Next-generation sequencing
NLS: Nuclear localization signal
NMD: Nonsense-mediated decay
NPC: Nuclear pore complex
pAS: Polyadenylation site
P-body: Processing bodies
PET: Positron emission tomography
PML: Promyelocytic leukemia protein
poly(A) tail: Polyadenosine tail
PrLD: Prion-like domain
PTM: Post-translational modification
RBP: RNA-binding protein
RNA: Ribonucleic acid
RNA-Seq: RNA sequencing
RRM: RNA Recognition Motif
SG: Stress granule
snRNPs: Small nuclear ribonucleoproteins
SNP: Single nucleotide polymorphism
SR proteins: Serine/arginine-rich proteins
sTDP-43: Short TDP-43
TAR: Trans-activation response
tRNA: Transfer RNA
[UG]_n_/UG: Uracil-guanine
UPR: Unfolded protein response
UTR: Untranslated region

## Glossary of terms

Antisense Oligonucleotides (ASO): Short, synthetic, single-stranded oligodeoxynucleotides which bind target mRNA and subsequently modify expression patterns
Amyotrophic lateral sclerosis (ALS): Neurodegenerative disease characterized by motor neuron degeneration and progressive muscle weakness. Now characterized as part of ALS-FTD disease continuum
CFTR minigene splicing assay: Assay used to detect TDP-43 functionality. Inclusion of exon 9 in CFTR mRNA is TDP-43 sensitive, with increased exon inclusion occurring with TDP-43 dysregulation
Cryptic exon (CE): Intronic region of DNA aberrantly included in mRNA
Frontotemporal dementia (FTD): Neurodegenerative disease characterized by degeneration of the frontal and/or temporal lobe. Now characterized as part of ALS-FTD disease continuum
Heterogeneous nuclear ribonucleoprotein (hnRNP): Large family of RNA-binding proteins composed of RNA-protein complexes
Liquid-liquid phase separation (LLPS): Process within cells in which molecules separate from cytoplasm through phase separation
Membraneless organelles (MLOs): Cellular compartments/substructures lacking a lipid membrane and often formed through LLPS. They include Cajal bodies, nucleoli, paraspeckles, P-bodies, promyelocytic leukemia (PML) protein bodies, and stress granules
Nuclear localization signal (NLS): Amino acid sequence within a protein that targets it to the nucleus
Polyadenylation (polyA): Process of adding a tail composed of adenine nucleotides to the polyA site (pAS) of pre-mRNA when forming mRNA. pre-mRNA with multiple pAS options can undergo alternative polyA (APA)
Post-translational modification (PTM): Translated proteins can be chemically modified at specific amino acids using functional groups or cleavage. These PTMs can significantly modify protein function and include acetylation, cleavage, phosphorylation, phosphomimetics, sumoylation, and ubiquitination
RAVER1: Regulatory splicing repressor protein
RNA-binding protein (RBP): Proteins that bind to RNA
RNA recognition motif (RRM): Protein domain capable of recognizing and binding RNA
Transposon: Non-coding DNA segment capable of moving between locations within the genome
U7smOPT: U7 small nuclear RNA molecule modified to specifically target known sequences and modify splicing patterns
